# Liuwei Dihuang Decoction Alleviates Cognitive Dysfunction in Mice With D-Galactose-Induced Aging by Regulating Lipid Metabolism and Oxidative Stress *via* the Microbiota-Gut-Brain Axis

**DOI:** 10.3389/fnins.2022.949298

**Published:** 2022-07-01

**Authors:** Baiyan Liu, Bowei Chen, Jian Yi, Hongping Long, Huiqiao Wen, Fengming Tian, Yingfei Liu, Lan Xiao, Lisong Li

**Affiliations:** ^1^The First Affiliated Hospital, Hunan University of Chinese Medicine, Changsha, China; ^2^Hunan Academy of Chinese Medicine, Changsha, China; ^3^College of Pharmacy, Hunan University of Chinese Medicine, Changsha, China; ^4^College of Information Science and Engineering, Hunan University of Chinese Medicine, Changsha, China

**Keywords:** Liuwei Dihuang decoction, aging, cognitive function, microbiota-gut-brain axis, metabolomics, lipid metabolism, oxidative stress

## Abstract

**Background:**

Aging is an important cause of cognitive dysfunction. Liuwei Dihuang decoction (LW), a commonly applied Chinese medicine formula, is widely used for the treatment of aging-related diseases in China. Previously, LW was confirmed to be effective in prolonging life span and reducing oxidative stress in aged mice. Unfortunately, the underlying mechanism of LW remains unclear. The aim of this study was to interpret the mechanism by which LW alleviates cognitive dysfunction related to aging from the perspective of the microbiota-gut-brain axis.

**Method:**

All C57BL/6 mice (*n* = 60) were randomly divided into five groups: the control, model, vitamin E (positive control group), low-dose LW and high-dose LW groups (*n* = 12 in each group). Except for those in the control group, D-galactose was subcutaneously injected into mice in the other groups to induce the aging model. The antiaging effect of LW was evaluated by the water maze test, electron microscopy, 16S rRNA sequencing, combined LC–MS and GC–MS metabolomics, and ELISA.

**Results:**

Liuwei Dihuang decoction ameliorated cognitive dysfunction and hippocampal synaptic ultrastructure damage in aging mice. Moreover, LW decreased *Proteobacteria* abundance and increased gut microbiota diversity in aging mice. Metabolomic analysis showed that LW treatment was associated with the significantly differential abundance of 14 metabolites, which were mainly enriched in apelin signaling, sphingolipid metabolism, glycerophospholipid and other metabolic pathways. Additionally, LW affected lipid metabolism and oxidative stress in aging mice. Finally, we also found that LW-regulated microbial species such as *Proteobacteria* and *Fibrobacterota* had potential relationships with lipid metabolism, oxidative stress and hippocampal metabolites.

**Conclusion:**

In brief, LW improved cognitive function in aging mice by regulating lipid metabolism and oxidative stress through restoration of the homeostasis of the microbiota-gut-brain axis.

## Introduction

Currently, the proportion of the world’s population that is elderly is growing rapidly. It is estimated that by 2050, the world’s elderly population will increase from 841 million in 2013 to 2 billion, representing 21% of the world’s population (Partridge et al., 2020). Cognitive impairment accompanies aging and becomes increasingly evident with age. At present, cognitive impairment has become a major problem that plagues the physical and mental health of elderly individuals. The hippocampus mainly regulates learning and memory, and the occurrence of cognitive impairment is closely related to functional changes in the hippocampus (Bettio et al., 2017; Solé et al., 2017). Maintaining the normal physiological function of the hippocampus can effectively alleviate the cognitive dysfunction caused by aging.

Liuwei Dihuang decoction (LW), a commonly applied traditional Chinese medicine formula, has been widely used for centuries in China for the treatment of aging-related diseases, and its efficacy has been validated by evidence-based medicine (Chen B. et al., 2019). Previous studies have found that LW can improve spatial learning ability by increasing neurogenesis in the dentate gyrus in rats (Lee et al., 2005). It also improves age-related learning and memory decline by inducing long-term potentiation (LTP) of hippocampal neurons (Huang et al., 2012). In addition, LW has been found to prolong the life span of elderly mice and reduce the oxidative stress state (Chen W. et al., 2019). Unfortunately, the mechanism of LW in the treatment of aging-related cognitive dysfunction remains unclear.

The gut is considered an important organ for regulating body function and promoting longevity due to its functions in immunity and nutrient intake (Qin et al., 2010). When the body enters the aging period, the intestinal barrier, absorption and immune function change, and various external factors can lead to the destruction of the intestinal microecological balance (Biagi et al., 2010). Studies have found that there are pathways connecting nerves in the gut and brain in the human body, which are closely related to gut microbes, and this connection is called the microbiota-gut-brain axis (Shabbir et al., 2021). The gut microbiota is an important player in bidirectional communication between the gut and the brain and can have a major impact on the body’s neurological function, not only by neurotransmitter secretion but also by immune and metabolic regulation (Dinan and Cryan, 2017; Bambury et al., 2018; Osadchiy et al., 2019). Therefore, restoring intestinal homeostasis and delaying the aging process are of great significance for improving the quality of life of the elderly population.

Metabolomics is mainly the study of small molecule metabolites as substrates and products of various metabolic pathways and can comprehensively reveal the changes occurring in organisms during the treatment of diseases (Baker, 2011). However, due to technical limitations, there is currently no technology that can describe all possible compounds in the body. Liquid chromatography–mass spectrometry (LC–MS) has a wide molecular weight range, with high sensitivity and simple preprocessing methodology, and is suitable for the detection of compounds with poor volatility, medium or strong polarity, and large molecular weight. Gas chromatography-MS (GC–MS) is suitable for the detection of highly volatile, small molecular weight and polar compounds (Zeki et al., 2020).

In this study, a combined LC–MS and GC–MS whole-spectrum metabolomics method was used to analyze the effect of LW on the metabolic profile of the hippocampus of mice with D-galactose-induced aging, and 16S ribosomal RNA (16S rRNA) sequencing was used to interpret the mechanism by which LW improves age-related cognitive dysfunction from the perspective of the microbiota-gut-brain axis.

## Materials and Methods

### Animals

Sixty male C57BL/6 mice with body weights of 18–22 g were purchased from Gempharmatech Co., Ltd. (Jiangsu, China) and housed in the specific pathogen-free (SPF) animal room of the First Affiliated Hospital of Hunan University of Chinese Medicine at a temperature of 22–26°C and a humidity of 45–55%. The animals were housed in groups of 5/cage. Distilled water and feed were freely provided, with natural light and bedding changed every other day. The Ethics Committee of Laboratory Animal Studies of the First Affiliated Hospital of Hunan University of Chinese Medicine approved all the experimental protocols (No. ZYFY20210710).

### Preparation of Liuwei Dihuang Decoction

Liuwei Dihuang decoction is composed of six herbs (Table 1), all of which were purchased from the First Affiliated Hospital of Hunan University of Chinese Medicine and qualified by the herbal medicinal botanist Hongping Long.

**TABLE 1:** Components of the Liuwei Dihuang decoction (LW).

Scientific name	Chinese name	English name	Part used	Origin	Batch number	Weight
*Rehmannia glutinosa* (Gaertn.) DC.	Shu Di Huang	Radix Rehmanniae	Root	Henan	2009063	24 g
*Cornus officinalis* Siebold & Zucc.	Shan Zhu Yu	Comus Officinalis	fruit	Henan	TH20111101	12 g
*Dioscorea oppositifolia* L.	Shan Yao	Rhizoma Dioscoreae	Root	Henan	CK20113001	12 g
*Paeonia suffruticosa* Andr.	Dan Pi	Paeoniaceae	Root bark	AnHui	NG20121102	9 g
*Alisma orientalis*(Sam.)Juzep.	Ze Xie	Alismatis	Tuber	SiChuan	CK20121503	9 g
*Wolfiporia extensa* (Peck) Ginn*s*	Fu Ling	Poria Cocos	Rhizome	Hunan	CK20120802	9 g

As in a previous study (Chen W. et al., 2019), *Rehmannia glutinosa* (Gaertn.) DC., *Cornus officinalis* Siebold & Zucc., *Dioscorea oppositifolia* L., *Paeonia suffruticosa* Andr., *Alisma orientalis* (Sam.) Juzep. and *Wolfiporia extensa* (Peck) Ginns were mixed at a ratio of 8:4:4:3:3:3. Distilled water at a 5× volume was then added for 1 h, after which the mixture was boiled for 2 h at 100°C and filtered with three layers of gauze. After washing with distilled water three times, the filtrate was extracted again by the same method. After combining the filtrate from the two extractions, the solution was concentrated to 2 g of crude drug/ml using a rotary evaporator.

### Main Reagents

Vitamin E soft capsules (H20003539) were purchased from Zhejiang Pharmaceutical Co., Ltd. (Hangzhou, China); D-galactose (v900922) was purchased from Sigma–Aldrich (Shanghai, China); mouse adiponectin ELISA kit (EK0596) was purchased from Boster (Wuhan, China); mouse apolipoprotein E (ApoE) ELISA kit (ab215086) and mouse free fatty acid (FFA) assay kit (ab65341) were purchased from Abcam (Cambridge, United Kingdom); and superoxide dismutase (SOD) assay kit (20190412), glutathione peroxidase (GSH-Px) assay kit (20190309), and malondialdehyde (MDA) assay kit (20190315) were purchased from Jiancheng (Nanjing, China).

### The UPLC-Q-TOF/MS for Quality Control of Liuwei Dihuang Decoction

As in our previous study (Chen et al., 2022), LW was analyzed by ultra-performance liquid chromatography coupled with quadrupole time-of-flight mass spectrometry (UPLC-Q-TOF/MS). In short, 10 mL of concentrated LW solution was placed in a 50 mL conical flask, and 30 mL of 70% methanol was added; LW was dissolved by ultrasonic vibration for 30 min. After standing, 2 mL of the solution was centrifuged at 8,000 r/min for 5 min, and then the supernatant was filtered through a 0.22 μm microporous membrane and placed in an injection bottle. The chromatography and MS conditions were the same as those previously reported (Chen et al., 2022).

### Experiment Design

**FIGURE 1:**
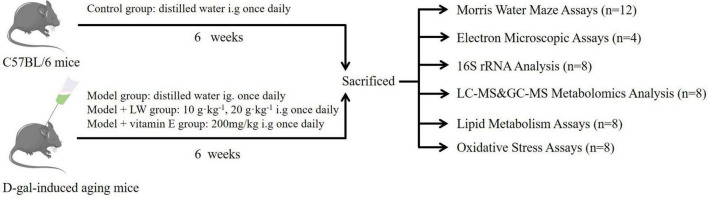
Experimental design of intervention study of Liuwei Dihuang decoction (LW).

A total of 60 mice were randomly divided into five groups after adaptive feeding: the control group, model group, vitamin E (200 mg⋅kg^–1^) group [positive control group, vitamin E has been used as a positive control in a large number of studies related to aging (Zeng et al., 2022)], low-dose LW (LW-L) (10 g⋅kg^–1^) group and high-dose LW (LW-H) (20 g⋅kg^–1^) group. Except for those in the control group, the mice in the other groups were subcutaneously injected with 100 mg⋅kg^–1^ D-galactose (Ali et al., 2015) once per day for 6 consecutive weeks, and subcutaneous injection of D-galactose is a commonly used method to replicate aging models (Huang X. et al., 2020). The control group mice were injected with the same volume of normal saline. After conducting the Morris water maze (MWM) test on the 6th week, we anesthetized all mice by intraperitoneal injection of 1% sodium pentobarbital. The eyeballs were removed, and the collected blood was centrifuged to obtain serum. Then, the contents of the cecum were collected under aseptic conditions, and the hippocampus of the mice was collected and placed on ice. All samples were stored at −80°C until further use. Eight mice from each group were randomly selected for 16S rRNA sequencing of cecal contents, metabolomics, and lipid metabolism and oxidative stress analysis, and the remaining four mice were analyzed by transmission electron microscopy (TEM), as shown in Figure 1.

### Morris Water Maze Test

The MWM was used to detect the learning and memory ability of mice and evaluate their cognitive function. According to previous reports (Hui et al., 2017), a black circular pool (depth 60 cm, diameter 150 cm) was used, the platform was fixed in the second quadrant, tap water was added to the pool, the water level was 2 cm higher than the platform, and the water temperature was controlled at 22–24°C. Mice were placed with their head toward the wall of the pool, and water points were randomly selected, with a detection time of 60 s. The time required for mice to find the submerged platform time was recorded, and if the platform was found within 60 s, the animal was allowed to stay on the platform for 10 s to rest; if the platform was not found, the animal was guided to the platform and left for 10 s. Each animal was trained three times daily, with a 15–20 min interval between each session, for 5 days. A video tracking system recorded animal location, swimming distance and time. On the 6th day, the platform was removed, the animals were put into the water from the opposite side of the original platform quadrant, and the latency and times of animals crossing the original platform quadrant within 60 s were recorded.

### Detection of the Synaptic Ultrastructure in the Hippocampus

Synaptic ultrastructure detection by TEM was performed as described previously (Sheng et al., 2020). Briefly, specimens that had been fixed for 48 h before TEM were postfixed in 1% osmic acid at room temperature for 2 h and dehydrated stepwise by an ethanol gradient and again by acetone. Tissues were first infiltrated overnight in solutions with different ratios of acetone to embedding medium, and then the tissues were polymerized in pure embedding medium at 60°C for 48 h for embedding. The tissue was cut into 60–80 nm ultrathin sections, stained with uranium acetate and lead citrate, and dried overnight at room temperature. The presynaptic membrane, postsynaptic membrane and synaptic vesicles were observed by TEM.

### 16S rRNA Analysis

Total genomic DNA was extracted using DNA Extraction Kit following the manufacturer’s instructions. The concentration of DNA was verified with a NanoDrop and agarose gel electrophoresis. The genomic DNA was used as a template for PCR amplification with barcoded primers and Tks Gflex DNA Polymerase (Takara). The primer sequences used to amplify the V3-V4 region were 343F: TACGGRAGGCAGCAG and 798R: AGGGTATCTAATCCT.

Then, the purified PCR products were quantified by a Qubit. The samples were mixed equally according to the concentration of PCR products and sequenced using an Illumina NovaSeq6000. Microbial community structure was assessed by alpha diversity and beta diversity analyses. In addition, phylogenetic investigation of communities by reconstruction of unobserved states (PICRUSt), the Kyoto Encyclopedia of Genes and Genomes (KEGG), and other functional spectrum databases were used for functional group prediction based on the 16S rDNA gene sequence. Library sequencing and data processing were conducted by OE Biotech Co., Ltd. (Shanghai, China).

### Metabolomics Analysis

#### LC-MS Analysis

First, 30 mg of hippocampal tissue was accurately weighed into a 1.5 ml EP tube with an internal standard (20 μl). Next, 600 μL of methanol-water (V:V = 4:1) was added. Then, two small steel beads were added and, after precooling in a −20°C freezer for 2 min, the sample was placed in a grinder for grinding (60 Hz, 2 min). Subsequently, the sample was submitted to ultrasonic extraction with an ice water bath for 10 min. Next, the sample was allowed to stand at −20°C for 2 h. Finally, the sample was centrifuged for 10 min (13,000 rpm, 4°C). Then, 150 μL of the supernatant was aspirated with a syringe, and the organic phase was filtered through a 0.22 μm pinhole filter, transferred to an LC injection vial, and stored at −80°C until LC–MS analysis was performed. The chromatography and MS analysis conditions and data processing methods are described in Supplementary Material 1.

#### GC-MS Analysis

Similar to the above steps, first, 30 mg of hippocampal tissue was accurately weighed into a 1.5 ml EP tube with an internal standard (20 μl). Then, 600 μL of methanol-water (V:V = 4:1) was added. Second, two small steel beads were added, and after precooling in a −20°C freezer for 2 min, the sample was placed in a grinder for grinding (60 Hz, 2 min). Third, 120 μL chloroform was added, and the sample was rotated for 2 min and then submitted to ultrasonic extraction in an ice water bath for 10 min. Fourth, the sample was allowed to stand at −20°C for 30 min. Subsequently, the sample was centrifuged at low temperature for 10 min (13,000 rpm, 4°C); 150 μL of supernatant was transferred into a standard glass flask. Next, the sample was dried with a centrifugal concentrator dryer. Then, 80 μL of methoxylamine hydrochloride pyridine solution (15 mg/mL) was added to the standard glass vials, and the vials were shaken for 2 min and then shaken in an incubator at 37°C for 90 min for the oxime reaction. After the samples were removed, 50 μL of bis(trimethylsilyl)trifluoroacetamide (BSTFA) (containing 1% chlorotrimethylsilane) derivatizing reagent and 20 μL of n-hexane were added, 10 kinds of internal standards (10 μL) were added, and the samples were shaken by vortexing for 2 min and reacted at 70°C for 60 min. After the samples were removed the heat block, they were placed at room temperature for 30 min for GC-MS analysis, and the data processing methods are shown in Supplementary Material 2. Both LC–MS and GC–MS analyses were performed by OE Biotech Co., Ltd. (Shanghai, China).

### Lipid Metabolism Assays

Serum levels of adiponectin, ApoE and FFA were determined using commercial kits according to the manufacturer’s instructions, and the absorbance was read at 450 nm (adiponectin and ApoE) and 570 nm (FFA).

### Oxidative Stress Detection

The hippocampal tissue was removed from the refrigerator, rinsed with precooled normal saline, cut into pieces with sterile ophthalmic scissors and added to normal saline at a m (tissue): V (normal saline) ratio = 1 g:9 mL (the sample was maintained on ice for the whole time). The sample was homogenized by an automatic homogenizer (intermittently once every 3 s to prevent excessive heat generation) to make a 10% mass fraction of homogenate, which was centrifuged at 1,000 × *g* (4°C) for 15 min. The supernatant was collected, and an appropriate amount of physiological saline was added to dilute the sample to an appropriate concentration. The instructions of the kit were strictly followed to detect SOD and GSH-Px activity and MDA content in serum and brain tissue.

### Statistical Analysis

GraphPad Prism 8.0.2 statistical analysis software was used for statistical analysis of the data. Metrology data are presented as the mean plus or minus the standard error ($\bar{x}\pm$s). Multiple comparisons among experiments were performed by one-way analysis of variance (ANOVA) with the least significant difference (LSD) test for multiple comparisons, and *P* < 0.05 was considered to indicate statistical significance.

In the 16S rRNA analysis, the operational taxonomic units (OTUs) were subjected to alpha diversity and beta diversity analysis. Communities or species that significantly differentially affected sample division were estimated using linear discriminant analysis (LDA) (Wang et al., 2022). For metabolomic analysis, orthogonal partial least squares discriminant analysis (OPLS-DA) was used to screen differentially abundant metabolites. Variable importance of projection (VIP) > 1 and *P* < 0.05 were used as thresholds to identify differentially abundant metabolites. To prevent overfitting, we used sevenfold cross validation and 200-response permutation testing (RPT) to assess the quality of the model (Wang et al., 2022). Finally, association analysis was performed using the Spearman correlation analysis method (Lu et al., 2022).

## Results

### Chemical Composition Analysis of Liuwei Dihuang Decoction

**FIGURE 2:**
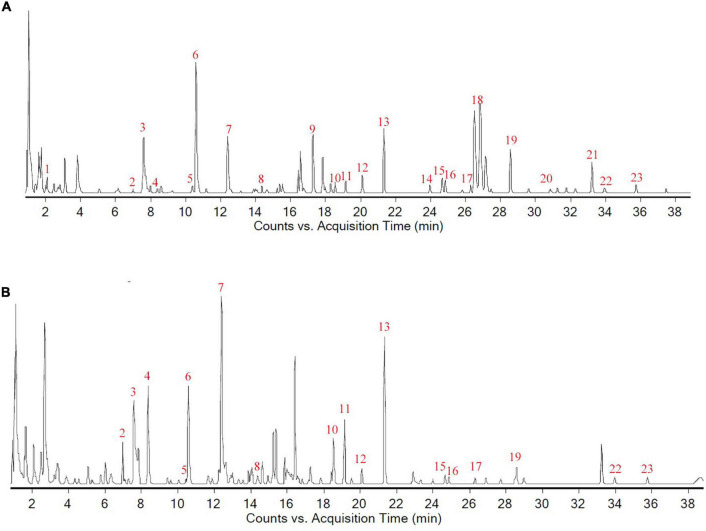
Chemical composition analysis of Liuwei Dihuang decoction. **(A)** The total composition chromatogram (TCC) in positive ion mode; **(B)** The TCC in negative ion mode.

The retention time (RT) and mass spectrometric data of each chemical component in LW were obtained after UPLC-Q-TOF-MS detection, and 23 components, including morroniside, loganin and paeonol, were resolved from the collected data, as shown in Figure 2 and Supplementary Table 1.

### Effects of Liuwei Dihuang Decoction on Cognitive Function and Hippocampal Synaptic Ultrastructure in Aging Mice

**FIGURE 3:**
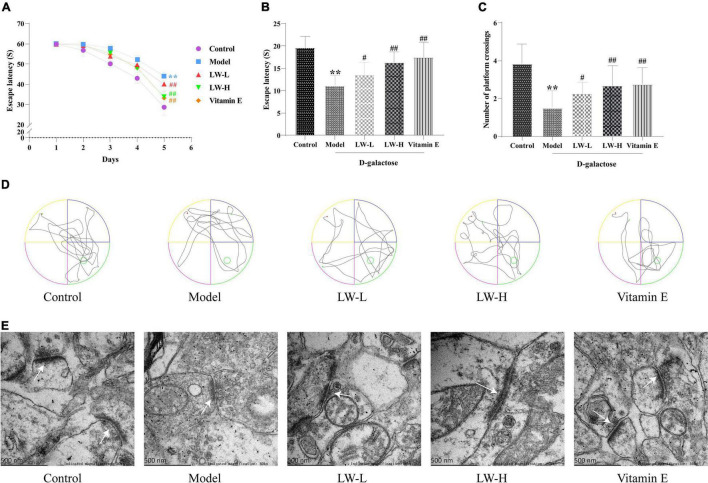
Effects of Liuwei Dihuang decoction on cognitive function and hippocampal synaptic ultrastructure in aging mice. **(A)** Navigation test. **(B)** Spatial exploration. **(C)** Platform crossing. **(D)** MWM representative figures. **(E)** Ultrastructure of hippocampal synapses. **p < 0.01 vs. Control group. ^#^*p* < 0.05, ^##^*p* < 0.01 vs. Model group.

In the navigation test, with the increase in training time and sessions, the time for mice in each group to find the platform tended to decrease. Compared with that of mice in the control group, the escape latency of mice in the model group was significantly prolonged (*P <* 0.01), and the latency of mice in the LW-L, LW-H, and vitamin E groups was significantly shortened compared with that of mice in the model group (*P* < 0.01, Figure 3A). The results of the spatial exploration test showed that compared with the control group mice, the model group mice also spent less time in the target quadrant (*P* < 0.01) and had significantly fewer platform crossings (*P* < 0.05), indicating that D-galactose injection induced cognitive dysfunction. Interestingly, LW improved D-galactose injection-induced cognitive impairment in a dose-dependent manner. Compared with those of mice in the model group, the time in the target quadrant (*P* < 0.05 or *P* < 0.01) and the number of platform crossings (*P* < 0.05 or *P* < 0.01) of mice in the LW-L, LW-H, and vitamin E groups were significantly increased (Figures 3B–D). These results suggest that LW can improve cognitive impairment in aging mice.

Transmission electron microscopy was used to assess the ultrastructure of hippocampal synapses. We found that the synaptic structure of the hippocampal neurons in the control group was intact, and the presynaptic membrane, synaptic cleft, postsynaptic membrane and postsynaptic dense material were clearly visible. Compared with those of the control group, the synaptic structure of the model group was blurred, the postsynaptic dense material was sparse, and the boundary between the anterior and posterior membranes was unclear. Compared with those of the model group, the synaptic structure of the LW-L, LW-H, and vitamin E groups was clearer, the boundaries of the anterior and posterior membranes were clear, and the postsynaptic dense matter increased, as shown in Figure 3E. It is suggested that LW can improve the damage to hippocampal synapses in aging mice.

### Effects of Liuwei Dihuang Decoction on the Gut Microbiota of Aging Mice

**FIGURE 4:**
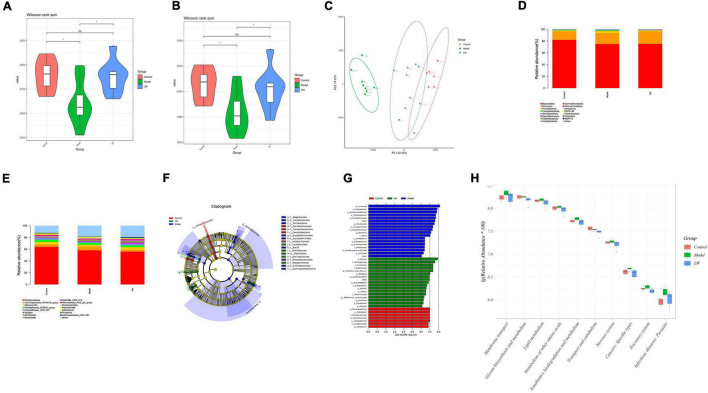
Effects of Liuwei Dihuang decoction on the gut microbiota of aging Mice. **(A)** Chao 1 index. **(B)** Observed species index. **(C)** PCoA analysis. **(D)** Relative abundance of gut microbiota (phylum level). **(E)** Relative abundance of gut microbiota (genus level). **(F)** Cladogram of LEfSe analysis. **(G)** LDA of LEfSe analysis. **(H)** PICRUSt2 analysis.

Based on the above MWM test results, the control, model and LW-H groups were selected for subsequent 16S rRNA and metabolomics analyses. Analysis of the alpha diversity, calculated using the Chao 1 index and observed species index, showed that the number and diversity of microbiota constituents in the model group relative to those in the control group decreased (*P* < 0.05), as shown in Figure 4A. After treatment with LW administration, the number and diversity of intestinal microbiota returned to their normal levels (*P* < 0.05), as shown in Figure 4B. In addition, beta diversity analysis was also utilized to assess differences between microbial communities. Principal coordinate analysis (PCoA) based on the weighted UniFrac distance showed that the microbial composition and structure of the model group and the control group were significantly different, and the LW group had a similar trend to the control group, suggesting that LW could partially restore the gut microbial composition of aging mice, as shown in Figure 4C.

To understand the effect of LW on the gut microbiota, we analyzed the relative abundance of gut microbiota constituents in the different groups. At the phylum level, aging mice showed a decreased relative abundance of *Bacteroidota* and an increased abundance of *Firmicutes* and *Proteobacteria*. In contrast, LW reduced the levels of *Proteobacteria* in aging mice (Figure 4D). At the genus level, the abundances of *Muribaculaceae*, *Alloprevotella*, *Prevotellaceae_UCG-001*, *Bacteroides* and *Clostridia_UCG-014* were decreased, and the abundances of *Lachnospiraceae_NK4A136_group* and *Parabacteroides* were increased in aging mice. LW treatment significantly increased the abundances of *Alloprevotella*, *Prevotellaceae_UCG-001*, *Bacteroides*, and *Clostridia_UCG-014* and decreased the abundances of *Lachnospiraceae_NK4A136_group* and *Parabacteroides*, as shown in Figure 4E.

Linear discriminate analysis effect size (LEfSe) analysis was applied to identify key microbiota members differentially represented in LW-treated mice. The dominant flora constituents in the LW group were *Clostridiacea*e and *Lactobacillales* at the order level, *Oscillospiraceae* and *Clostridiaceae* at the class level, and *Alistipes* and *Roseburia* at the genus level, as shown in Figures 4F,G.

Finally, to determine whether taxonomic changes in the gut microbiota affected its function, we performed functional prediction based on representative sequences with PICRUSt2. Based on a comparison with the control group, there were significant differences (*P* < 0.05) in glycan biosynthesis and metabolism, lipid metabolism, metabolism of other amino acids, exogenous compound biodegradation and metabolism, transport and catabolism, and the nervous system. LW reversed the above changes in pathways (*P* < 0.05), as shown in Figure 4H.

### Effects of Liuwei Dihuang Decoction on the Metabolic Profile of Hippocampal Tissue in Aging Mice

**FIGURE 5:**
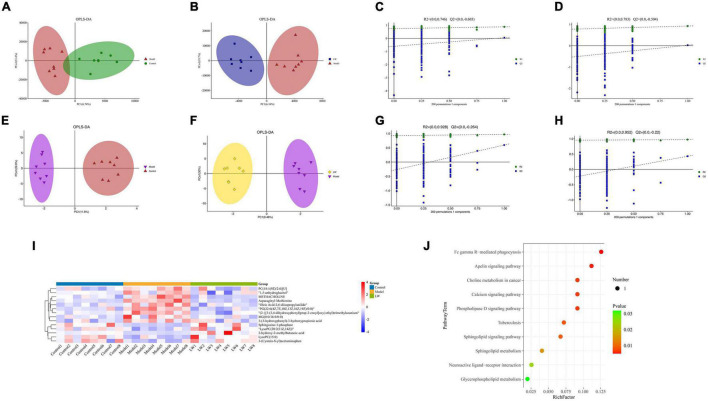
Effects of LW on the metabolic profile of hippocampal tissue in aging mice. **(A)** OPLS-DA of LC-MS (Model vs. Control). **(B)** OPLS-DA of LC-MS (LW vs. Model). **(C)** Permutation of OPLS-DA model (Model vs. Control). **(D)** Permutation of OPLS-DA model (LW vs. Model). **(E)** OPLS-DA of GC-MS (Model vs. Control). **(F)** OPLS-DA of GC-MS (LW vs. Model). **(G)** Permutation of OPLS-DA model (Model vs. Control). **(H)** Permutation of OPLS-DA model (LW vs. Model). **(I)** Differentially abundant metabolites. **(J)** Analysis of metabolic pathway enrichment.

The effects of LW on the metabolites in the hippocampal tissue of aging mice were first analyzed by LC–MS. OPLS-DA was used to distinguish the overall differences in the metabolic profiles between the groups, as shown in Figures 5A,B. The differences among the control group, model group and LW group were obvious. In addition, to prevent model overfitting, 7-fold cross validation and 200-RPT methods were used to examine the quality of the model. The results showed that compared with the control group, the model group had R2 = 0.737 and Q2 = −0.708 (Figure 5C). Moreover, compared with the model group, the LW group had R2 = 0.793 and Q2 = −0.471 (Figure 5D), suggesting that the OPLS-DA model is stable and has good predictive ability. Finally, according to the screening thresholds of VIP > 1 and *p* < 0.05, the metabolites with differential abundances between different groups were determined. There were 47 metabolites with differential abundances between the model group and the control group and 30 metabolites with differential abundances between the LW group and the model group (Supplementary Table 2).

Subsequently, we analyzed the effects of LW on the metabolites in the hippocampal tissue of aging mice by GC–MS. GC–MS is able to detect compounds with strong volatility, small molecular weight, and low polarity and can be used as a complement to LC–MS for identifying thermally stable compounds (Zeki et al., 2020). OPLS-DA was used to distinguish the overall differences in metabolic profiles among the groups, as shown in Figures 5E,F, which were obvious among the control, model and LW groups. Evaluation of the OPLS-DA model showed that the model group had R2 = 0.928 and Q2 = −0.264 compared with the control group (Figure 5G). Moreover, compared with the model group, the LW group had R2 = 0.952 and Q2 = −0.22 (Figure 5H), suggesting that the OPLS-DA model is stable and has good predictive ability. Finally, we found a total of 53 metabolites with differential abundances between the model group and the control group and 22 metabolites with differential abundances between the LW group and the model group, as shown in Supplementary Table 3.

By integrating data from the dual-platform metabolomic analysis, we found that LW reversed the changes in the levels of 14 differentially abundant metabolites in the model group, of which five metabolites were depleted in the model group and enriched in the LW group, and the other nine metabolites were enriched in the model group and depleted in the LW group, as shown in Figure 5I and Table 2. Using the KEGG database, we analyzed the metabolic pathways in which the above 14 metabolites were involved. Finally, we found that apelin signaling, calcium signaling, phospholipase D signaling, sphingolipid metabolism, neuroactive ligand–receptor interaction and glycerophospholipid metabolism were significantly enriched metabolic pathways (*p* < 0.05), as shown in Figure 5J. Excitingly, we found that as determined by metabolomics, the significant enrichment of sphingolipid metabolism and glycerophospholipid metabolism closely resembled the changes in metabolic pathways such as lipid metabolism in the gut microbiota that were predicted by PICRUSt based on 16S rRNA sequencing data. This alignment suggests that the effects of LW intervention on the gut microbiota and hippocampal metabolites may be related.

**TABLE 2:** Identification and variation tendency of 14 differential metabolites by LC-MS & GC-MS.

Metabolites	Compound ID	Mode	Formula	Model vs. Control		LW vs. Model	
					
				VIP	*P*-value	VIP	*P*-value
LysoPC(20:2(11Z,14Z))	HMDB0010392	LC-MS	C_28_H_54_NO_7_P	1.97653516	0.009151234	2.257710481	0.017475092
LysoPC(15:0)	HMDB0010381	LC-MS	C_23_H_48_NO_7_P	1.072222462	0.006313074	1.190052623	0.034020136
3-(Cystein-S-yl)acetaminophen	HMDB0240217	LC-MS	C_11_H_14_N_2_O_4_S	1.007448543	0.03183613	1.207377077	0.009769263
2-hydroxy-2-methylbutanoic acid	HMDB0001987	GC-MS	/	1.792910311	6.49489E−06	2.572525386	0.040679786
3-(3-hydroxyphenyl)-3-hydroxypropionic acid	HMDB0002643	GC-MS	/	1.997405882	0.004959811	1.58286374	0.041028306
PC(18:1(9Z)/2:0)[U]	39642	LC-MS	C_28_H_54_NO_8_P	1.273559624	0.009100443	1.174542693	0.040487874
(2-{[3-(3,4-dihydroxyphenyl)prop-2-enoyl]oxy}ethyl) trimethylazanium	HMDB0136312	LC-MS	C_14_H_20_NO_4_	1.282963036	0.000837746	1.688485234	0.001014712
1,5-anhydroglucitol	HMDB0002712	GC-MS	/	1.316636006	0.012282553	2.310523814	0.049125615
PG(22:6(4Z,7Z,10Z,13Z, 16Z,19Z)/0:0)	LMGP04050016	LC-MS	C_28_H_45_O_9_P	2.006290372	0.000168751	2.574115085	2.89809E−05
METHACHOLINE	43545	LC-MS	C_8_H_17_NO_2_	4.259755705	0.00245965	5.736589467	0.000250805
Sphingosine-1-phosphate	3891	LC-MS	C_18_H_38_NO_5_P	1.64865281	0.047778962	1.663805976	0.048089092
Oleic Acid-2,6-diisopropylanilide	64645	LC-MS	C_30_H_51_NO	1.021481093	0.003157587	1.074957858	0.003608528
MG(0:0/16:0/0:0)	HMDB0011533	LC-MS	C_19_H_38_O_4_	1.188356611	0.036927406	1.290358375	0.044092221
Asparaginyl-Methionine	HMDB0028737	LC-MS	C_9_H_17_N_3_O_4_S	1.800887048	1.06379E−05	2.219478238	5.13366E−06

### Effects of Liuwei Dihuang Decoction on Lipid Metabolism and Oxidative Stress in Aging Mice

**FIGURE 6:**
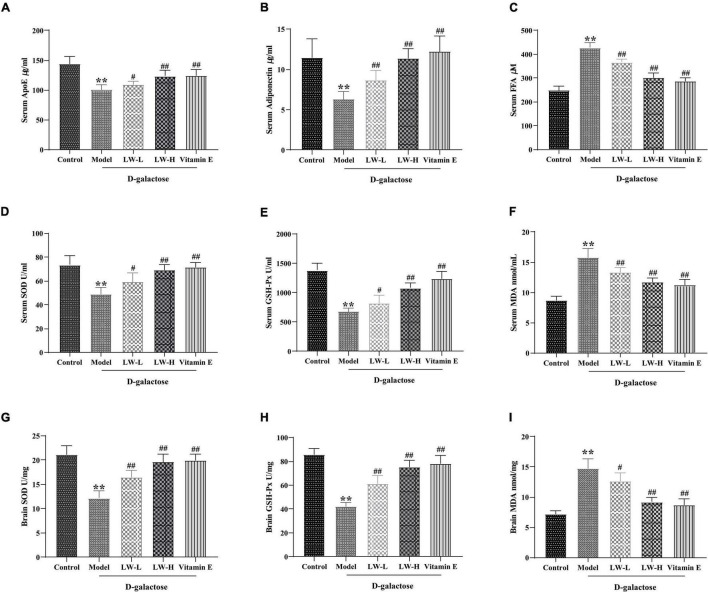
Effects of Liuwei Dihuang decoction on lipid metabolism and oxidative stress in aging mice. **(A)** ApoE. **(B)** Adiponectin. **(C)** FFA. **(D)** SOD in serum. **(E)** GSH-Px in serum. **(F)** MDA in serum. **(G)** SOD in brain tissue. **(H)** GSH-Px in brain tissue. **(I)** MDA in brain tissue. ^**^p < 0.01 vs. Control group. ^#^*p* < 0.05, ^##^*p* < 0.01 vs. Model group.

The above results of 16S rRNA sequencing and metabolomic analysis of the hippocampus suggest that LW may play a therapeutic role by affecting lipid metabolism in aging mice. Therefore, we further studied the effect of LW on the lipid metabolism-related factors ApoE, adiponectin and FFA in the serum of rapidly aging mice. As shown in Figures 6A–C, compared with those in the control group, the ApoE and adiponectin contents in the model group were significantly decreased (*p* < 0.01), and the FFA content was significantly increased (*p* < 0.01), while LW and vitamin E treatment reversed the levels of these factors (*p* < 0.05 or *p* < 0.01). This finding suggested that LW can affect lipid metabolism in aging mice.

Disturbances in lipid metabolism are often accompanied by oxidative stress, which is often also an important risk factor for aging and cognitive dysfunction (Morgan et al., 2016). Therefore, we examined the effects of LW on the activities of SOD, GSH-Px and MDA content in the serum and brain tissue of aging mice. As shown in Figures 6D–I, compared with the control group, the MDA content in the serum and brain tissue of the mice in the model group was significantly increased (*P* < 0.05), and the activities of SOD and GSH-Px were significantly decreased (*P* < 0.01), indicating that the antioxidant capacity of serum and brain tissue of D-galactose-induced aging mice was significantly reduced. LW and vitamin E reversed the above changes (*P* < 0.05 or *P <* 0.01), suggesting that LW could improve the antioxidant capacity of model mice.

### Relationship Between Gut Microbes and Hippocampal Metabolites, Lipid Metabolism, and Oxidative Stress

**FIGURE 7:**
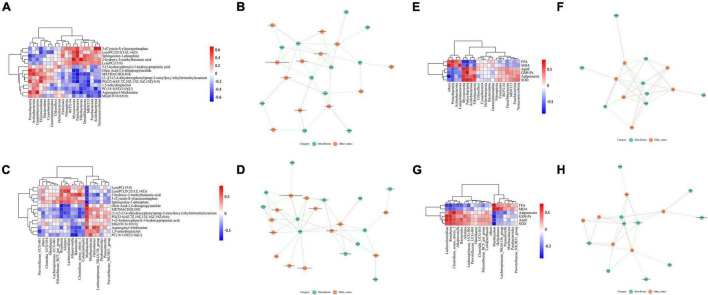
Relationship between gut microbes and hippocampal metabolites, lipid metabolism and oxidative stress. **(A,B)** Correlation of microbiota related to differentially abundant metabolites (phylum level). **(C,D)** Correlation of microbiota related to differentially abundant metabolites (genus level). **(E,F)** Correlation of microbiota with factors related to lipid metabolism and oxidative stress (phylum level). **(G,H)** Correlation of microbiota with factors related to lipid metabolism and oxidative stress (genus level).

Next, we analyzed the potential relationship between the differentially abundant gut microbiota constituents and differentially abundant metabolites in the hippocampus. As shown in Figures 7A,B, at the phylum level, *Proteobacteria* was positively correlated with oleic acid-2,6-diisopropylanilide, methacholine, PG [22:6(4Z,7Z,10Z,13Z,16Z,19Z)/0:0] and asparaginyl-methionine (*p* < 0.05). At the genus level, *Roseburia* and *Lachnoclostridium* were positively correlated with lipid metabolites such as sphingosine-1-phosphate and lysophosphatidylcholine lysoPC [20:2 (11z, 14z)] (*P* < 0.05), and *Muribaculum* was significantly negatively correlated with lipid metabolites such as sphingosine-1-phosphate, lysoPC (15:0) and lysoPC [20:2 (11z, 14z)] (*P* < 0.05, *P* < 0.01 or *P* < 0.001), as shown in Figures 7C,D.

Similarly, we analyzed the correlation of the microbiota with factors related to lipid metabolism and oxidative stress in serum. At the phylum level, *Proteobacteria* was positively correlated with FFA and MDA (*p* < 0.05 or *p* < 0.01), and negatively correlated with ApoE, GSH-Px, adiponectin, and SOD (*p* < 0.05); *Fibrobacterota* was positively correlated with ApoE, GSH-Px, adiponectin and SOD were positively correlated (*p* < 0.05), and negatively correlated with FFA and MDA (*p* < 0.05), as shown in Figures 7E,F. At the genus level, *Muribaculum* was positively correlated with FFA and MDA (*p* < 0.01 or *p* < 0.001), and negatively correlated with ApoE, GSH-Px, adiponectin, and SOD (*p* < 0.01 or *p* < 0.001); *Roseburia* was positively correlated with ApoE, GSH-Px, adiponectin and SOD were positively correlated (*p* < 0.05 or *p* < 0.01), and negatively correlated with FFA and MDA (*p* < 0.05 or *p* < 0.01), as shown in Figures 7G,H. Thus, these findings revealed potential interactions between LW-regulated microbial species and lipid metabolism and oxidative stress.

## Discussion

At present, the proportion of the elderly population in the world is growing rapidly, and the challenges associated with aging have become an international problem. Aging is an irreversible natural law, and researchers cannot prevent aging, although they can delay it. The multitarget and multilinking mechanism of traditional Chinese medicine is an important direction for aging delay therapeutic development. In this study, we found that LW can improve cognitive function in aging mice and improve the synaptic structure of the hippocampus. Subsequently, by 16S rRNA sequencing, we found that LW improved gut microbiota diversity in rapidly aging mice, and subsequent metabolomic analysis revealed that LW could alter the levels of endogenous metabolites in the hippocampus of aging mice. Notably, both 16S rRNA sequencing and metabolomic functional enrichment analyses identified lipid metabolism as a target of LW. In subsequent explorations, we confirmed that LW could affect lipid metabolism and oxidative stress in aging mice.

The gut microbiota is considered to be the host’s “second genome,” which plays important roles in maintaining the body’s homeostasis and in the occurrence and development of cognitive dysfunction (Li et al., 2019; Liu et al., 2021b). Studies have shown that the gut microecological characteristics of elderly individuals include lower diversity (Bunker et al., 2019), depletion of *Bacteroidota*, and enrichment of *Proteobacteria* and *Firmicutes* (Luo et al., 2020). Other studies have pointed out that increased *gram-negative bacteria* in the intestinal flora can lead to increased secretion of proinflammatory cytokines, and these products can reach the central nervous system (CNS) through the circulation to promote neuroinflammatory responses (Wang and Quinn, 2010). Moreover, signaling molecules secreted by the gut microbiota are transferred *via* the lymphatic and systemic circulation throughout the CNS where they then modulate brain plasticity and cognitive function (de Haas and van der Eijk, 2018). Our study showed that LW treatment was able to improve the alpha diversity index of the gut microbiota and reduce the levels of *Proteobacteria*. *Proteobacteria* is the largest phylum of bacteria and accounts for a small proportion in the healthy adult gut, and its enrichment is considered a marker of gut dysbiosis (Shin et al., 2015). Previous studies have shown that *Proteobacteria* is closely related to cognitive impairment in elderly individuals (Manderino et al., 2017), and that reducing the level of *Proteobacteria* can improve cognitive function in aging mice (Yang et al., 2020). At the genus level, we found that the abundance of *Alloprevotella* decreased and that of *Parabacteroides* increased in aging mice, and LW reversed these changes. *Alloprevotella* is a beneficial bacterium and is closely related to lipid metabolism in aging mice (Wu et al., 2022), and elevating the abundance of *Alloprevotella* can improve memory function in mice (Liu et al., 2021a). Studies have shown that *Parabacteroides* is an independent risk factor for mild cognitive impairment in elderly individuals (Khine et al., 2020) and that a high abundance of *Parabacteroides* can exacerbate neurodegeneration (Blacher et al., 2019). Further LEfSe analysis revealed that *Lactobacillales* (order), *Clostridiaceae* (class), *Alistipes* (genus), and *Roseburia* (genus) were the dominant microbiota constituents in the LW group. *Lactobacillales* regulates intestinal microbes and enhances immunity and antioxidation (De-Filippis et al., 2020). Previous studies have confirmed that *Lactobacillales* can improve the gut microbiota in aging rats (Hor et al., 2019), inhibit oxidative stress (Wang et al., 2021), and improve cognitive function in aged mice (Ni et al., 2019). Higher *Clostridiaceae* abundance correlates with better “attention continuity” (Komanduri et al., 2021). Previous studies have shown that a high abundance of *Alistipe*s can effectively suppress intestinal inflammation and oxidative stress (Wan et al., 2021). *Roseburia* produces short-chain fatty acids, affects intestinal motility, has anti-inflammatory properties and is considered the cornerstone of gut biodiversity (Tamanai-Shacoori et al., 2017). Finally, we found that glycan biosynthesis and metabolism, lipid metabolism, metabolism of other amino acids, exogenous compound biodegradation and metabolism, transport and catabolism, and the nervous system might be the main metabolic pathways in the differential flora through PICRUSt2 analysis.

Increasing evidence suggests that metabolic changes associated with the gut microbiota are important factors associated with various diseases (Liu et al., 2022). Mammalian humoral and tissue metabolomes are greatly influenced by the microbiome, and many health-related metabolites are considered “mammal-microbial cometabolites” (Heinken and Thiele, 2015; Chen et al., 2018). Based on the combined GC–MS and LC–MS/MS whole-spectrum metabolomics platform used in this study, we systematically analyzed endogenous metabolites in the hippocampus of aging mice treated with LW and identified 14 differentially abundant metabolites as potential LW metabolic markers for effects on aging mice. These potential metabolic markers mainly include metabolites closely related to lipid metabolism, such as phosphatidylcholine (PC) and lysoPC. LysoPCs are the most biologically active lysophospholipids; they are key signaling molecules in cell and tissue metabolism and are involved in plasma membrane formation (Shindou et al., 2013), cell growth and death (Makide et al., 2009). In addition, sphingosine-1-phosphate, a potential metabolic marker, has the ability to modulate the stress resistance, proliferation, differentiation and maturation phenotype of cells in the nervous system (Czubowicz et al., 2019). Studies have confirmed that sphingosine-1-phosphate content in the hippocampus decreases gradually with age (Couttas et al., 2018), and that activation of sphingosine-1-phosphate reduces Aβ deposition and improves cognitive function in Alzheimer’s disease (AD) rats (Wang and Yang, 2021). KEGG enrichment analysis revealed that apelin signaling, calcium signaling, phospholipase D signaling, sphingolipid metabolism, neuroactive ligand–receptor interaction and glycerophospholipid metabolism may be the main metabolic pathways involved in the mechanism of action of LW. Apelin, a cytokine produced and secreted by adipocytes, has been shown to be involved in processes such as the regulation of fluid homeostasis, food intake, cell proliferation, and angiogenesis (Zhou et al., 2018). Moreover, apelin has been recently identified as an adipokine involved in energy metabolism, which can act together with leptin and adiponectin on glucose metabolism and lipid metabolism (Bertrand et al., 2015). In an apelin knockout mouse model, the aging rate was accelerated. When apelin content was restored, it was found that not only the vitality of the mice was enhanced but also the behavior and circadian rhythm phenotypes were restored, suggesting that apelin may be an antiaging factor (Rai et al., 2017). Sphingolipids are lipids that are highly enriched in the CNS and are involved in membrane formation and signal transduction, cell proliferation, apoptosis, migration and invasion, inflammation and nervous system development. Disorders of sphingolipid metabolism mediate the occurrence and development of many neurological diseases, such as Parkinson’s disease, multiple sclerosis and AD (Alaamery et al., 2021).

Notably, the enrichment analysis based on 16S rRNA sequencing and differential metabolomics in the hippocampus suggested that LW may play a therapeutic role by affecting lipid metabolism in the body. Some studies have suggested that abnormal lipid metabolism is closely related to cognitive dysfunction (Bowers et al., 2020). Therefore, we further explored the effect of LW on the lipid metabolism-related factors ApoE, adiponectin, and FFA in the serum of rapidly aging mice. The results suggested that LW treatment can affect the levels of ApoE, adiponectin and FFA in the serum of aging mice. ApoE is mainly produced in the liver, is able to transport lipids, and plays a central role in lipid metabolism (Yin and Wang, 2018). There is recent evidence for a similar role for ApoE in the brain (Holtzman et al., 2012): as the major CNS apolipoprotein, ApoE is responsible for regulating much of brain lipid metabolism, in particular the transfer of cholesterol and phospholipids from glial cells to neurons (Hudry et al., 2019). Furthermore, loss of ApoE disrupts the blood–brain barrier (BBB) in aging mice (Mulder et al., 2001) and leads to cognitive dysfunction (Zerbi et al., 2014) and cerebrovascular dysfunction (Bell et al., 2012). Adiponectin is a plasma protein capable of crossing the BBB, exerting its modulation and signaling effects through its receptors (Bloemer et al., 2018). Adiponectin has been found to regulate glucose metabolism in hippocampal neurons, increasing rates of glucose uptake, glycolysis and ATP production (Cisternas et al., 2019). St Studies have shown that circulating adiponectin levels are decreased in mild cognitive impairment and AD (Teixeira et al., 2013) and that tail vein injection of adiponectin-overexpressing endothelial progenitor cells can improve cognitive function in aging rats (Huang J. et al., 2020). FFA is closely related to lipid metabolism, and clinical studies have shown that high FFA plasma concentrations are associated with reduced cognitive function (Holloway et al., 2011). Disturbances in lipid metabolism are often accompanied by oxidative stress (Zarrouk et al., 2020), which is often also an important risk factor for aging and cognitive dysfunction (Mecocci et al., 2018). Abnormal oxidative stress can cause protein degeneration, lipid peroxidation, DNA damage and other physiological function changes in cells or tissues, leading to apoptosis and tissue damage, and is a major risk factor for neurodegenerative conditions such as aging (Vatner et al., 2020). Our research shows that the MDA content in the serum and hippocampus of aging mice is significantly increased and that the total SOD (T-SOD) and GSH-Px activities are significantly reduced. LW delays aging and inhibits oxidative damage. Our study showed that the MDA content in the serum and hippocampus of aging mice was significantly increased and that the T-SOD and GSH-Px activities were significantly decreased. Intervention with LW reversed the above changes, suggesting that LW can improve the antioxidant capacity of cells, delay aging and inhibit oxidative damage.

Recently, the microbiota-gut-brain axis has gained extensive attention as a channel for communication and physiological regulation (Li et al., 2020). Experimental evidence suggests that the gut microbiota can alter the levels of multiple cytokines, which in turn can have a significant effect on several brain functions (Giau et al., 2018). *Bacteria* have recently been identified in the brains of AD patients, suggesting that the microbiota may be a contributing factor to related neuroinflammation (Emery et al., 2017). Probiotics can modulate gut microbiota dysbiosis and microbiota–gut–brain axis deficits to improve cognitive dysfunction in aged mice (Yang et al., 2020). Furthermore, gut microbiome activity may promote abnormal lipid deposition and oxidation that damage the brain (Shao et al., 2020). Regulating the composition of the intestinal flora and the abundance of beneficial bacteria (including *Enterorhabdu*s, *Clostridium*, *Bifidobacterium*, and *Parvibacter*) were able to slow down D-gal-induced oxidative stress damage and apoptosis of neurons (Liu et al., 2021c). We found that *Proteobacteria* was positively correlated with FFA and MDA (*p* < 0.05 or *p* < 0.01) and negatively correlated with ApoE, GSH-Px, adiponectin, and SOD (*p* < 0.05), suggesting that the high abundance of *Proteobacteria* may aggravate lipid deposition and promote oxidative stress. Conversely, the high abundance of *Fibrobacterota* may inhibit oxidative stress. Based on the above studies, we speculated that LW might regulate the levels of *Proteobacteria* and *Fibrobacterota* to affect lipid metabolism and oxidative stress to improve age-related cognitive dysfunction, but the mechanism remains to be further investigated.

Notably, there are some limitations of this study. We did not use germ-free mice or fecal bacterial transplantation to determine which flora constituents are related to the therapeutic effect of LW. In addition, the findings still need to be clinically validated, and the specific mechanisms of differentially abundant bacteria or metabolites and lipid metabolism were not explored in depth and need further investigation.

## Conclusion

In summary, this study confirmed the ability of LW to improve cognitive function and hippocampal synaptic structure in aging mice, modulate the gut microbial composition and hippocampal metabolic profile in aging mice, and modulate lipid metabolism and oxidative stress. Moreover, this study also showed that the changes in the *Proteobacteria* and *Fibrobacterota* induced by LW may have a potential link with lipid metabolism and oxidative stress, which deserves further investigation.

## Data Availability Statement

The datasets presented in this study can be found in online repositories. The names of the repository/repositories and accession number(s) can be found in the article/Supplementary Material.

## Ethics Statement

The animal study was reviewed and approved by the Ethics Committee of Laboratory Animal Studies of The First Affiliated Hospital of Hunan University of Chinese Medicine approved all the experimental protocols (No. ZYFY20210710).

## Author Contributions

BL designed the experiments, analyzed the data, and prepared the manuscript. BC and JY performed the experiments, analyzed the data, and prepared the manuscript. HL, HW, FT, and YL performed the experiments. LX and LL optimized the language of the manuscript. All authors confirmed the final manuscript.

## Conflict of Interest

The authors declare that the research was conducted in the absence of any commercial or financial relationships that could be construed as a potential conflict of interest.

## Publisher’s Note

All claims expressed in this article are solely those of the authors and do not necessarily represent those of their affiliated organizations, or those of the publisher, the editors and the reviewers. Any product that may be evaluated in this article, or claim that may be made by its manufacturer, is not guaranteed or endorsed by the publisher.

## Supplementary Material

Supplementary Table 1 Chemical composition analysis of LW.

Supplementary Table 2 Differential metabolites identified by LC-MS.

Supplementary Table 3 Differential metabolites identified by GC-MS.

Supplementary Material 1 Chromatographic and MS analytical conditions and data processing methods for LC-MS.

Supplementary Material 2 Chromatographic and MS analytical conditions and data processing methods for GC-MS.
