# Supplementary material for: Liuwei Dihuang Decoction Alleviates Cognitive Dysfunction in Mice With D-Galactose-Induced Aging by Regulating Lipid Metabolism and Oxidative Stress via the Microbiota-Gut-Brain Axis
Source: Front Neurosci. 2022 Jul 1;16:949298. doi: 10.3389/fnins.2022.949298 (PMC9283918; doi:10.3389/fnins.2022.949298)
Supplement: Supplementary Table 1 — Chemical composition analysis of LW. [file Table_1.DOCX]

| Number | RT | Positive Ion Mode | Negative Ion Mode | Molecular Mass | Molecular Formula | Name | Deviation |
| --- | --- | --- | --- | --- | --- | --- | --- |
| 1 | 2.085 | M+H 127.0376 | / | 126.0304 | C_6_ H_6_O_3_ | 5-hydroxymethylfurfural | (+) 11.96 |
| 2 | 6.988 | M+Na 399.1252 | M-H 375.1301 | 376.36 | C_16_H_24_O_10_ | Loganic acid | (+) 4.01 (-) -1.05 |
| 3 | 7.616 | M+Na 429.1270 | M+COOH 451.1462 | 406.3817 | C_17_H_26_O_11_ | Morroniside | (+) 0.68 |
| 4 | 8.383 | M+H 497.0945 | M-H 495.1513 | 496 | C_23_H_28_O_12_ | Oxypaeoniflora | (+) -4.15 (-) -0.83 |
| 5 | 10.393 | M+H 483.1462 | M-H 481.1114 | 482.1392 | C_31_H_46_O_4_ | Polyporenic acid C | (+) -1.51 |
| 6 | 10.614 | M+Na 413.1369 | M+COOH 435.1513 | 390.38 | C_17_H_26_O_10_ | Loganin | (+) 2.75 |
| 7 | 12.387 | M+Na 503.1504 | M+COOH 525.162 | 480.45 | C_23_H_28_O_11_ | Paeoniflorin | (+) 4.34 |
| 8 | 14.374 | M+H 447.1272 | M+COOH 491.1198 | 446.404 | C_22_H_22_O_10_ | Syringaresino | (+) 3.07 |
| 9 | 17.275 | M+H 397.304 | / | 396.2948 | C_28_H_44_O | Ergosterol | / |
| 10 | 18.543 | M+NH_4_ 618.2174 | M-H 599.1769 | 600.57 | C_30_H_35_NO_13_ | Benzoyloxypaeoniflorin | (+) 0.98 (-) 0.42 |
| 11 | 19.149 | M+NH_4_ 618.2181 | M-H 599.1780 | 600.57 | C_30_H_35_NO_13_ | MudanpiosideC | (+) 0.47 (-) -1.47 |
| 12 | 20.105 | M+H 285.0747 | M-H 283.0618 | 284.0684 | C_16_H_12_O_5_ | Calycosin | (+) 3.1 (-) -2.06 |
| 13 | 21.336 | M+NH_4_ 602.2216 | M+COOH 629.1885 | 584.57 | C_30_H_35_NO_12_ | Benzoylpaeoniflorin | (+) 3.04 |
| 14 | 23.886 | M+H 167.0698 | M-H 164.8361 | 166.18 | C_9_H_10_O_3_ | Paeonol | (+) 2.39 |
| 15 | 24.668 | M+H 269.0799 | M-H 267.0664 | 268.0736 | C_10_H_13_N_5_O_4_ | Adenosine | (+) 3.07 (-) -0.59 |
| 16 | 24.849 | N+H 529.3506 | M+COOH+H2O 591.3536 | 528.3435 | C_32_H_48_O_6_ | Alisol C 23-acetate | (+) 2.96 |
| 17 | 26.292 | N+H 529.3508 | M-H 527.3377 | 528.3438 | C_33_H_52_O_5_ | Pachymic acid | (+) 2.57 |
| 18 | 26.514 | M+NH_4_ 274.2749 | / | 256.42 | C_16_H_35_NO_2_ | Palmitic acid | (+) -1.98 |
| 19 | 28.563 | M+H 487.3402 | M+COOH 485.3273 | 486.693 | C_30_H_46_O_5_ | Alisol C | (+) 3.26 |
| 20 | 30.843 | M+NH_4_ 302.3033 | / | 284.48 | C_18_H_39_NO_2_ | stearic acid | (+) 6.16 |
| 21 | 33.229 | M+H 473.3606 | / | 472.3535 | C_30_ H_48_O_4_ | Alisol B | (+) -4.67 |
| 22 | 33.948 | M+Na 555.3643 | M+COOH 577.3749 | 532.762 | C_32_ H_48_O_4_ | Alisol A,24-acetate | (+) 2.49 |
| 23 | 35.754 | M+H 515.3713 | M+COOH+H2O 577.3742 | 514.74 | C_32_H_50_O_5_ | Alisol B 23-acetate | (+) 2.92 |
