# Supplementary material for: Liuwei Dihuang Decoction Alleviates Cognitive Dysfunction in Mice With D-Galactose-Induced Aging by Regulating Lipid Metabolism and Oxidative Stress via the Microbiota-Gut-Brain Axis
Source: Front Neurosci. 2022 Jul 1;16:949298. doi: 10.3389/fnins.2022.949298 (PMC9283918; doi:10.3389/fnins.2022.949298)
Supplement: Supplementary Material 1 — Chromatographic and MS analytical conditions and data processing methods for LC-MS. [file Data_Sheet_1.PDF]

LC-MS 报告-英文部分

上海鹿明生物科技有限公司

Shanghai Lu-Ming Biotech Co.,Ltd., Shanghai, China

注：本英文说明仅供参考使用，请勿直接摘抄到文章里面。

如需要请根据自己的标准进行编辑后，再放在文章里面。

1. 样本前处理

1.1 植物样本

Chemicals

All chemicals and solvents were analytical or HPLC grade. Water, methanol, acetonitrile, formic acid were purchased from Thermo Fisher Scientific(Thermo Fisher Scientific, Waltham, MA, USA). L-2-chlorophenylalanine was from Shanghai Heng chuang Bio-technology Co., Ltd. (Shanghai, China).

Sample Preparation

** mg accurately weighed sample was transferred to a 1.5 mL Eppendorf tube. Two small steel balls were added to the tube. **μL of L-2-chlorophenylalanine (0.3 mg / mL) dissolved in methanol as internal standard and ** mL mixture of methanol and water (7/3, vol/vol) were added to each sample, samples were placed at -20 °C for 2min. Then grinded at 60 HZ for 2 min, and the whole samples were extracted by ultrasonic for 30 min in ice-water bath, then placed at -20°C for 20 min. Samples were centrifuged at 4°C (13,000 rpm) for 10 min prior to decanting of ** μL supernatants from each tube were collected using crystal syringes, filtered through 0.22 μm microfilters and transferred to LC vials. The vials were stored at -80°C until LC -MS analysis.

QC were prepared by mixing aliquot of the all samples to be a pooled sample.

1.2 细胞/菌体

Chemicals

All chemicals and solvents were analytical or HPLC grade. Water, methanol, acetonitrile, formic acid were purchased from Thermo Fisher Scientific(Thermo Fisher Scientific, Waltham, MA, USA). L -2-chlorophenylalanine was from Shanghai Heng chuang Bio-technology Co., Ltd. (Shanghai, China). Chloroform was from Titan Chemical Reagent Co., Ltd. (Shanghai, China).

Sample Preparation

** mL of methanol: water (4/1, vol/vol) were added to each sample, then transformed to a 4 mL glass vial. ** μL of chloroform were added to each aliquot, dispersing sample by pipette. Using ultrasonic homogenizer to breaking up the cells for 6 min at 500 w. All of the mixtures of each sample were transferred to 1.5 mL Eppendorf tubes, ** μL of L-2-chlorophenylalanine (0.3 mg / mL) dissolved in methanol as internal standard， then extracted by ultrasonication for 20 min in ice-water bath. The extract was centrifuged at 4°C (13,000 rpm) for 10 min . ** mL of supernatant in a glass vial was dried in a freeze concentration centrifugal dryer . ** μL mixture of methanol and water (1/4, vol/vol) were added to each sample, samples vortexed for 30 s, extracted by ultrasonic for 3 min in ice-water bat, then placed at -20°C for 2 h. Samples were centrifuged at 4°C (13,000 rpm) for 10 min. The supernatants (** μL) from each tube were collected using crystal syringes, filtered through 0.22 μm microfilters and transferred to LC vials. The vials were stored at -80°C until LC -MS analysis.

QC samples were prepared by mixing aliquot of the all samples to be a pooled sample.

注：本英文说明仅供参考使用，请勿直接摘抄到文章里面。如需要请根据自己的标准进行编辑后，再放在文章里面。

1.3 培养液/发酵液

Chemicals

All chemicals and solvents were analytical or HPLC grade. Water, methanol, acetonitrile, formic acid were purchased from Thermo Fisher Scientific(Thermo Fisher Scientific, Waltham, MA, USA). L-2-chlorophenylalanine was from Shanghai Heng chuang Bio-technology Co., Ltd. (Shanghai, China).

Sample Preparation

** μL of sample were added to an 1.5 mL Eppendorf tube, then were dried in a freeze drier. ** μL of L-2-chlorophenylalanine (0.3 mg/mL) dissolved in methanol as internal standard， ** μL mixture of methanol and water (1/4, vol/vol) were added to each sample, samples vortexed for 30 s, extracted by ultrasonic for 3 min in ice-water bat, then placed at -20°C for 2 h. Samples were centrifuged at 4°C (13,000 rpm) for 10 min. The supernatants (** μL) from each tube were collected using crystal syringes, filtered through 0.22 μm microfilters and transferred to LC vials. The vials were stored at -80°C until LC -MS analysis.

QC samples were prepared by mixing aliquot of the all samples to be a pooled sample.

注：本英文说明仅供参考使用，请勿直接摘抄到文章里面。如需要请根据自己的标准进行编辑后，再放在文章里面。

1.4 血清

Chemicals

All chemicals and solvents were analytical or HPLC grade. Water, methanol, acetonitrile, formic acid were purchased from Thermo Fisher Scientific(Thermo Fisher Scientific, Waltham, MA, USA). L-2-chlorophenylalanine was from Shanghai Heng chuang Bio-technology Co., Ltd. (Shanghai, China).

Sample Preparation

Samples stored at -80 ℃ were thawed at room temperature. ** μL of sample was added to a 1.5 mL Eppendorf tube with ** μL of L-2-chlorophenylalanine (0.3 mg/mL) dissolved in methanol as internal standard, and the tube was vortexed for 10 s. Subsequently, ** μL of ice-cold mixture of methanol and acetonitrile (2/1, vol/vol) was added, and the mixtures were vortexed for 1 min, and the whole samples were extracted by ultrasonic for 10 min in ice-water bath，stored at -20 ℃ for 30 min. The extract was centrifuged at at 4°C (13,000 rpm) for 10 min. ** μL of supernatant in a glass vial was dried in a freeze concentration centrifugal dryer .** μL mixture of methanol and water (1/4, vol/vol) were added to each sample, samples vortexed for 30 s, extracted by ultrasonic for 3 min in ice-water bat, then placed at -20°C for 2 h. Samples were centrifuged at 4°C (13,000 rpm) for 10 min. The supernatants (** μL) from each tube were collected using crystal syringes, filtered through 0.22 μm microfilters and transferred to LC vials. The vials were stored at -80°C until LC -MS analysis.

QC samples were prepared by mixing aliquot of the all samples to be a pooled sample.

注：本英文说明仅供参考使用，请勿直接摘抄到文章里面。如需要请根据自己的标准进行编辑后，再放在文章里面。

1.5 尿液

Chemicals

All chemicals and solvents were analytical or HPLC grade. Water, methanol, acetonitrile, formic acid were purchased from Thermo Fisher Scientific(Thermo Fisher Scientific, Waltham, MA, USA). L -2-chlorophenylalanine was from Shanghai Heng chuang Bio-technology Co., Ltd. (Shanghai, China).

Sample Preparation

Samples stored at -80 ℃ were thawed at room temperature. ** μL of sample was added to a 1.5 mL Eppendorf tube with ** μL of L-2-chlorophenylalanine (0.3 mg/mL) dissolved in methanol as internal standard, and the tube was vortexed for 10 s. Subsequently, ** μL of ice-cold mixture of methanol and acetonitrile (2/1, vol/vol) was added, and the mixtures were vortexed for 1 min, and the whole samples were extracted by ultrasonic for 10 min in ice-water bath， stored at -20 ℃ for 30 min. The extract was centrifuged at at 4°C (13,000 rpm) for 10 min. ** μL of supernatant in a glass vial was dried in a freeze concentration centrifugal dryer .** μL mixture of methanol and water (1/4, vol/vol) were added to each sample, samples vortexed for 30 s, extracted by ultrasonic for 3 min in ice-water bat, then placed at -20°C for 2 h. Samples were centrifuged at 4°C (13,000 rpm) for 10 min. The supernatants (** μL) from each tube were collected using crystal syringes, filtered through 0.22 μm microfilters and transferred to LC vials. The vials were stored at -80°C until LC -MS analysis.

QC samples were prepared by mixing aliquot of the all samples to be a pooled sample.

注：本英文说明仅供参考使用，请勿直接摘抄到文章里面。如需要请根据自己的标准进行编辑后，再放在文章里面。

1.6 动物组织

Chemicals

All chemicals and solvents were analytical or HPLC grade. Water, methanol, acetonitrile, formic acid were purchased from Thermo Fisher Scientific(Thermo Fisher Scientific, Waltham, MA, USA). L -2-chlorophenylalanine was from Shanghai Heng chuang Bio-technology Co., Ltd. (Shanghai, China).

Sample Preparation

** mg accurately weighed sample was transferred to a 1.5 mL Eppendorf tube. Two small steel balls were added to the tube. **μL of L-2-chlorophenylalanine (0.3 mg / mL) dissolved in methanol as internal standard and ** mL mixture of methanol and water (4/1, vol/vol) were added to each sample. Samples were stored at -20 °C for 2 min and then grinded at 60 HZ for 2 min, and the whole samples were extracted by ultrasonic for 10 min in ice-water bath, stored at -20 °C for 30 min. The extract was centrifuged at at 4°C (13,000 rpm) for 10 min. ** μL of supernatant in a glass vial was dried in a freeze concentration centrifugal dryer .** μL mixture of methanol and water (1/4, vol/vol) were added to each sample, samples vortexed for 30 s, extracted by ultrasonic for 3 min in ice-water bat, then placed at -20°C for 2 h. Samples were centrifuged at 4°C (13,000 rpm) for 10 min. The supernatants (** μL) from each tube were collected using crystal syringes, filtered through 0.22 μm microfilters and transferred to LC vials. The vials were stored at -80°C until LC -MS analysis.

QC samples were prepared by mixing aliquot of the all samples to be a pooled sample.

2. 样品上机处理

注：本英文说明仅供参考使用，请勿直接摘抄到文章里面。如需要请根据自己的标准进行编辑后，再放在文章里面。

2.1 Waters-VION IMS QTOF/ACQUITY UPLC I-Class

ACQUITY UPLC I-Class system (Waters Corporation，Milford, USA) coupled with VION IMS QTOF Mass spectrometer(Waters Corporation，Milford, USA) was used to analyze the metabolic profiling in both ESI positive and ESI negative ion modes. An ACQUITY UPLC BEH C18 column (1.7 μm, 2.1 × 100 mm) were employed in both positive and negative modes. Water and Acetonitrile / Methanol 2/3(v/v), both containing 0.1% formic acid were used as mobile phases A and B, respectively. Linear gradient: 0 min, 1% B; 1min, 30% B; 2.5min, 60% B; 6.5min, 90% B; 8.5min, 100% B; 10.7 min, 100% B; 10.8 min, 1% B and 13min, 1%B. The flow rate was 0.4 mL/min and column temperature was 45 ℃. All the samples were kept at 4℃ during the analysis. The injection volume was 1 μL.

Data acquisition was performed in full scan mode (m/z ranges from 50 to 1000) combined with MSE mode, including 2 independent scans with different collision energies (CE) were alternatively acquired during the run. Parameters of mass spectrometry were as follows: a low-energy scan (CE 4eV), and a high-energy scan (CE ramp 20-45eV) to fragment the ions. Argon (99.999%) was used as collision-induced dissociation gas；scan time: 0.2s; interscan delay: 0.02s; capillary voltage: 2.5 kV; cone voltage: 40 V; source temperature: 115 ℃; desolvation gas temperature: 450 ℃; desolvation gas flow, 900 L/h.

The QCs were injected at regular intervals (every ** samples) throughout the analytical run to provide a set of data from which repeatability can be assessed.

2.2 Thermo Q-EXACTIVE plus/Dionex U3000 UHPLC

A Dionex Ultimate 3000 RS UHPLC fitted with Q-Exactive plus quadrupole-Orbitrap mass spectrometer equipped with heated electrospray ionization(ESI) source (Thermo Fisher Scientific, Waltham, MA, USA) was used to analyze the metabolic profiling in both ESI positive and ESI negative ion modes. An ACQUITY UPLC HSS T3 column (1.8 μm, 2.1 × 100 mm) were employed in both positive and negative modes. The binary gradient elution system consisted of (A) water (containing 0.1 % formic acid, v/v) and (B) acetonitrile (containing 0.1 % formic acid, v/v) and separation was achieved using the following gradient: 0 min, 5% B; 2min, 5% B; 4min, 25% B; 8min, 50% B; 10min, 80% B; 14min, 100% B; 15 min, 100% B; 15.1 min, 5% and 16 min, 5%B. The flow rate was 0.35 mL/min and column temperature was 45 °C. All the samples were kept at 4°C during the analysis. The injection volume was ** μL.

The mass range was from m/z 100 to 1,000. The resolution was set at 70,000 for the full MS scans and 17,500 for HCD MS/MS scans. The Collision energy was set at 10, 20 and 40 eV. The mass spectrometer operated as follows: spray voltage, 3,800 V (+) and 3,000 V (−); sheath gas flow rate, 35 arbitrary units; auxiliary gas flow rate, 8 arbitrary units; capillary temperature, 320°C; Aux gas heater temperature, 350°C; S-lens RF level, 50.

The QCs were injected at regular intervals (every ** samples) throughout the analytical run to provide a set of data from which repeatability can be assessed.

2.3 Thermo Q-EXACTIVE /Dionex U3000 UHPLC

A Dionex Ultimate 3000 RS UHPLC fitted with Q-Exactive quadrupole-Orbitrap mass spectrometer equipped with heated electrospray ionization(ESI) source (Thermo Fisher Scientific, Waltham, MA, USA) was used to analyze the metabolic profiling in both ESI positive and ESI negative ion modes. An ACQUITY UPLC HSS T3 column (1.8 μm, 2.1 × 100 mm) were employed in both positive and negative modes. The binary gradient elution system consisted of (A) water (containing 0.1 % formic acid, v/v) and (B) acetonitrile (containing 0.1 % formic acid, v/v) and separation was achieved using the following gradient: 0.01 min, 5% B; 2min, 5% B; 4min, 30% B; 8min, 50% B; 10min, 80% B; 14min, 100% B; 15 min, 100% B; 15.1 min, 5% and 18 min, 5%B. The flow rate was 0.35 mL/min and column temperature was 45 ℃. All the samples were kept at 4℃ during the analysis. The injection volume was ** μL.

The mass range was from m/z 100 to 1,000. The resolution was set at 70,000 for the full MS scans and 17,500 for HCD MS/MS scans. The Collision energy was set at 10, 20 and 40 eV. The mass spectrometer operated as follows: spray voltage, 3,800 V (+) and 3,200 V (−); sheath gas flow rate, 40 arbitrary units; auxiliary gas flow rate, 8 arbitrary units; capillary temperature, 320°C; Probe Heater Temperature, 350°C; S-lens RF level, 50.

The QCs were injected at regular intervals (every ** samples) throughout the analytical run to provide a set of data from which repeatability can be assessed.

注：本英文说明仅供参考使用，请勿直接摘抄到文章里面。如需要请根据自己的标准进行编辑后，再放在文章里面。

2.4 Thermo Q-EXACTIVE /Nexera UPLC

A Nexera UPLC system (Shimadzu Corporation, Japan) coupled with Q-Exactive quadrupole-Orbitrap mass spectrometer equipped with heated electrospray ionization(ESI) source (Thermo Fisher Scientific, Waltham, MA, USA) was used to analyze the metabolic profiling in both ESI positive and ESI negative ion modes. An ACQUITY UPLC HSS T3 column (1.8 μm, 2.1 × 100 mm) were employed in both positive and negative modes. The binary gradient elution system consisted of (A) water (containing 0.1 % formic acid, v/v) and (B) acetonitrile (containing 0.1 % formic acid, v/v) and separation was achieved using the following gradient: 0 min, 5% B; 2min, 5% B; 4min, 25% B; 8min, 50% B; 10min, 80% B; 14min, 100% B; 15 min, 100% B; 15.1 min, 5% and 16 min, 5%B. The flow rate was 0.35 mL/min and column temperature was 45 ℃. All the samples were kept at 4℃ during the analysis. The injection volume was ** μL.

The mass range was from m/z 125 to 1,000. The resolution was set at 70,000 for the full MS scans and 17,500 for HCD MS/MS scans. The Collision energy was set at 10, 20 and 40 eV. The mass spectrometer operated as follows: spray voltage, 3,500 V (+) and 3,500 V (−); sheath gas flow rate, 40 arbitrary units(+) and 35 arbitrary units(-); auxiliary gas flow rate, 10 arbitrary units(+) and 8 arbitrary units(-); capillary temperature, 320°C.

The QCs were injected at regular intervals (every ** samples) throughout the analytical run to provide a set of data from which repeatability can be assessed.

2.5 Thermo Q-EXACTIVE /AB ExionLc

A AB ExionLc system (AB SCIEX, Framingham, MA) coupled with Q-Exactive quadrupole-Orbitrap mass spectrometer equipped with heated electrospray ionization(ESI) source (Thermo Fisher Scientific, Waltham, MA, USA) was used to analyze the metabolic profiling in both ESI positive and ESI negative ion modes. An ACQUITY UPLC HSS T3 column (1.8 μm, 2.1 × 100 mm) were employed in both positive and negative modes. The binary gradient elution system consisted of (A) water (containing 0.1 % formic acid, v/v) and (B) acetonitrile (containing 0.1 % formic acid, v/v) and separation was achieved using the following gradient: 0 min, 5% B; 2min, 5% B; 4min, 25% B; 8min, 50% B; 10min, 80% B; 14min, 100% B; 15 min, 100% B; 15.1 min, 5% and 16 min, 5%B. The flow rate was 0.35 mL/min and column temperature was 45 ℃. All the samples were kept at 4℃ during the analysis. The injection volume was ** μL.

The mass range was from m/z 100 to 1,200. The resolution was set at 70,000 for the full MS scans and 17,500 for HCD MS/MS scans. The mass spectrometer operated as follows: spray voltage, 3,800 V (+) and 3,200 V (−); sheath gas flow rate, 40 arbitrary units; auxiliary gas flow rate, 10 arbitrary units; capillary temperature, 350°C.

The QCs were injected at regular intervals (every ** samples) throughout the analytical run to provide a set of data from which repeatability can be assessed.

注：本英文说明仅供参考使用，请勿直接摘抄到文章里面。如需要请根据自己的标准进行编辑后，再放在文章里面。

2.6 AB6600 plus/AB ExionLc

A AB ExionLc system (AB SCIEX, Framingham, MA) coupled with an AB SCIEX Triple TOF 5600 System (AB SCIEX, Framingham, MA) was used to analyze the metabolic profiling in both ESI positive and ESI negative ion modes. An ACQUITY UPLC HSS T3 column (1.8 μm, 2.1 × 100 mm) were employed in both positive and negative modes. The binary gradient elution system consisted of (A) water (containing 0.1 % formic acid, v/v) and (B) acetonitrile (containing 0.1 % formic acid, v/v) and separation was achieved using the following gradient: 0 min, 5% B; 2min, 5% B; 4min, 25% B; 8min, 50% B; 10min, 80% B; 14min, 100% B; 15 min, 100% B; 15.1 min, 5% and 16 min, 5%B. The flow rate was 0.35 mL/min and column temperature was 45 ℃. All the samples were kept at 4℃ during the analysis. The injection volume was ** μL.

Data acquisition was performed in full scan mode (m/z ranges from 100 to 1000) combined with IDA mode. Parameters of mass spectrometry were as follows: Ion source temperature, 550 °C (+) and 550 °C (−); ion spray voltage, 5500 V (+) and 4500 V (−); curtain gas of 35 PSI; nebulizer gas of 55 PSI; auxiliary gas of 55 PSI; declustering potential, 80V (+) and 80 V (−); collision energy, 10 eV (+) and 10 eV (−); and interface heater temperature, 550 °C (+) and 550 °C (−). For IDA analysis, range of m/z was set as 40–1000, the collision energy was 35 eV.

The QCs were injected at regular intervals (every ** samples) throughout the analytical run to provide a set of data from which repeatability can be assessed.

2.7 Waters-Xevo G2-XS QTof

ACQUITY UPLC system (Waters Corporation，Milford, USA) coupled with Xexo G2-XS Qtof Mass spectrometer(Waters Corporation，Milford, USA) was used to analyze the metabolic profiling in both ESI positive and ESI negative ion modes. An ACQUITY UPLC BEH C18 column (1.7 μm, 2.1 × 100 mm) were employed in both positive and negative modes. . The binary gradient elution system consisted of (A) water (containing 0.1 % formic acid, v/v) and (B) acetonitrile (containing 0.1 % formic acid, v/v) and separation was achieved using the following gradient: 0 min, 1% B; 1min, 5% B; 2min, 30% B; 3.5min, 60% B; 7.5min, 90% B; 9.5min, 100% B; 12.5min, 100% B; 12.7min, 1% B and 16min, 1%B. The flow rate was 0.4 mL/min and column temperature was 45 ℃. All the samples were kept at 4℃ during the analysis. The injection volume was 2 μL .

Data acquisition was performed in full scan mode (m/z ranges from 50 to 1000) combined with MSE mode , scan time , 0.1s; including 2 independent scans with different collision energies (CE) were alternatively acquired during the run. Parameters of mass spectrometry were as follows: a low-energy scan (CE 6eV), and a high-energy scan (CE ramp 20-35eV) to fragment the ions. Capillary voltage, 2 kV (negative mode) / 3 kV (positive mode); sampling cone, 40 V; source offset, 80 V; source temperature, 120 ℃; desolvation gas temperature, 450 ℃, desolvation gas flow, 800 L/h, and cone gas flow, 50 L/h.

The QCs were injected at regular intervals (every 10 samples) throughout the analytical run to provide a set of data from which repeatability can be assessed.

注：本英文说明仅供参考使用，请勿直接摘抄到文章里面。如需要请根据自己的标准进行编辑后，再放在文章里面。

3. 数据分析

注：本英文说明仅供参考使用，请勿直接摘抄到文章里面。如需要请根据自己的标准进行编辑后，再放在文章里面。

Data Preprocessing and Statistical Analysis

The original LC-MS data were processed by software Progenesis QI V2.3 (Nonlinear, Dynamics, Newcastle, UK) for baseline filtering, peak identification, integral, retention time correction, peak alignment, and normalization. Main parameters of 5 ppm precursor tolerance, 10 ppm product tolerance, and 5% product ion threshold were applied. Compound identification were based on precise mass-to-charge ratio (M/z), secondary fragments, and isotopic distribution using The Human Metabolome Database (HMDB), Lipidmaps (V2.3), Metlin, EMDB, PMDB, and self-built databases to do qualitative analysis.

The extracted data were then further processed by removing any peaks with a missing value (ion intensity = 0) in more than 50% in groups, by replacing zero value by half of the minimum value, and by screening according to the qualitative results of the compound. Compounds with resulting scores below 36 (out of 60) points were also deemed to be inaccurate and removed. A data matrix was combined from the positive and negative ion data.

The matrix was imported in R to carry out Principle Component Analysis (PCA) to observe the overall distribution among the samples and the stability of the whole analysis process. Orthogonal Partial Least-Squares-Discriminant Analysis (OPLS-DA) and Partial Least-Squares-Discriminant Analysis (PLS-DA) were utilized to distinguish the metabolites that differ between groups. To prevent overfitting, 7-fold cross-validation and 200 Response Permutation Testing (RPT) were used to evaluate the quality of the model.

Variable Importance of Projection (VIP) values obtained from the OPLS-DA model were used to rank the overall contribution of each variable to group discrimination. A two-tailed Student's T-test was further used to verify whether the metabolites of difference between groups were significant. Differential metabolites were selected with VIP values greater than 1.0 and p-values less than 0.05.
