# Supplementary material for: Liuwei Dihuang Decoction Alleviates Cognitive Dysfunction in Mice With D-Galactose-Induced Aging by Regulating Lipid Metabolism and Oxidative Stress via the Microbiota-Gut-Brain Axis
Source: Front Neurosci. 2022 Jul 1;16:949298. doi: 10.3389/fnins.2022.949298 (PMC9283918; doi:10.3389/fnins.2022.949298)
Supplement: Supplementary Material 2 — Chromatographic and MS analytical conditions and data processing methods for GC-MS. [file Data_Sheet_2.PDF]

GC-MS 报告-英文部分

上海鹿明生物科技有限公司

Shanghai Lu-Ming Biotech Co.,Ltd., Shanghai, China

注：本英文说明仅供参考使用，请勿直接摘抄到文章里面。

如需要请根据自己的标准进行编辑后，再放在文章里面。

1. 样本前处理

注：本英文说明仅供参考使用，请勿直接摘抄到文章里面。如需要请根据自己的标准进行编辑后，再放在文章里面。

1.1 植物样本

Chemicals

All chemicals and solvents were analytical or HPLC grade. Water, methanol were purchased from Thermo Fisher Scientific(Thermo Fisher Scientific, Waltham, MA, USA). Pyridine, n-hexane, methoxylamine hydrochloride（97%）, BSTFA with 1% TMCS were purchased from CNW Technologies GmbH (Düsseldorf, Germany). Chloroform was from Titan Chemical Reagent Co., Ltd. (Shanghai, China). L-2-chlorophenylalanine was from Shanghai Heng chuang Bio-technology Co., Ltd. (Shanghai, China).

Sample Preparation

** mg accurately weighed sample was transferred to a 1.5 mL Eppendorf tube. Two small steel balls were added to the tube. ** μL of cold methanol and ** μL of L-2-chlorophenylalanine (0.3 mg/mL) dissolved in methanol as internal standard were added to each sample, samples were placed at -20 ℃ for 2 min. Then grinded at 60 HZ for 2 min, and the whole samples were extracted by ultrasonic for 30 min in ice-water bath. ** μL of chloroform was added to the samples, and the mixtures were vortexed, ** μL water was added. Samples were vortexed again, and the whole samples were extracted by ultrasonic for 30 min in ice-water bath, then placed at -20°C for 20 min. Samples were centrifuged at 4°C (13,000 rpm) for 10 min . ** μL of supernatant in a glass vial was dried in a freeze concentration centrifugal dryer. QC sample was prepared by mixing aliquot of the all samples to be a pooled sample. An aliquot of the ** μL supernatant was transferred to a glass sampling vial for vacuum-dry at room temperature. And ** μL of 15 mg/mL methoxylamine hydrochloride in pyridine was subsequently added. The resultant mixture were vortexed vigorously for 2 min and incubated at 37 ℃ for 90 mi n. ** μL of BSTFA (with 1% TMCS) and ** μL n-hexane was added into the mixture, which was vortexed vigorously for 2 min and then derivatized at 70 ℃ for 60 min. The samples were placed at ambient temperat ure for 30 min before GC-MS analysis.

1.2 细胞/菌体

Chemicals

All chemicals and solvents were analytical or HPLC grade. Water, methanol were purchased from Thermo Fisher Scientific(Thermo Fisher Scientific, Waltham, MA, USA). Pyridine, n-hexane, methoxylamine hydrochloride（97%）, BSTFA with 1% TMCS were purchased from CNW Technologies GmbH (Düsseldorf, Germany). Chloroform was from Titan Chemical Reagent Co., Ltd. (Shanghai, China). L-2-chlorophenylalanine was from Shanghai Heng chuang Bio-technology Co., Ltd. (Shanghai, China).

Sample Preparation

** mL of methanol: water (4/1, vol/vol) were added to each sample, then transformed to a 4 mL glass vial. ** μL of chloroform were added to each aliquot, dispersing sample by pipette. Using ultrasonic homogenizer to breaking up the cells for 6 min at 500 w. All of the mixtures of each sample were transferred to 1.5 mL Eppendorf tubes, ** μL of L-2-chlorophenylalanine (0.3 mg / mL) dissolved in methanol as internal standard， then extracted by ultrasonication for 20 min in ice-water bath. The extract was centrifuged at 4°C (13,000 rpm) for 10 min . ** mL of supernatant in a glass vial was dried in a freeze concentration centrifugal dryer . QC sample was prepared by mixing aliquot of the all samples to be a pooled sample. An aliquot of the ** μL supernatant was transferred to a glass sampling vial for vacuum-dry at room temperature. And ** μL of 15 mg/mL methoxylamine hydrochloride in pyridine was subsequently added. The resultant mixture was vortexed vigorously for 2 min and incubated at 37 ℃ for 90 mi n. ** μL of BSTFA (with 1% TMCS) and ** μL n-hexane were added into the mixture, which was vortexed vigorously for 2 min and then derivatized at 70 ℃ for 60 min. The samples were placed at ambient temperat ure for 30 min before GC-MS analysis.

1.3 培养液/发酵液

Chemicals

All chemicals and solvents were analytical or HPLC grade. Acetonitrile, methanol were purchased from Thermo Fisher Scientific(Thermo Fisher Scientific, Waltham, MA, USA). Pyridine, n-hexane, methoxylamine hydrochloride（97%）, BSTFA with 1% TMCS were purchased from CNW Technologies GmbH (Düsseldorf, Germany). L-2-chlorophenylalanine was from Shanghai Heng chuang Bio-technology Co., Ltd. (Shanghai, China).

Sample Preparation

** μL of sample were added to an 1.5 mL Eppendorf tube, then were dried in a freeze drier. ** μL of L-2-chlorophenylalanine (0.3 mg/mL) dissolved in methanol as internal standard， ** μL mixture of methanol and acetonitrile (2/1, vol/vol) were added to each sample, samples vortexed for 30 s, extracted by ultrasonic for 3 min in ice-water bat, then placed at -20℃ for 20 h. Samples were centrifuged at 4℃ (13,000 rpm) for 10 min. ** mL of supernatant in a glass vial was dried in a freeze concentration centrifugal dryer . QC sample was prepared by mixing aliquot of the all samples to be a pooled sample. An aliquot of the ** μL supernatant was transferred to a glass sampling vial for vacuum-dry at room temperature. And ** μL of 15 mg/mL methoxylamine hydrochloride in pyridine was subsequently added. The resultant mixture was vortexed vigorously for 2 min and incubated at 37 ℃ for 90 mi n. ** μL of BSTFA (with 1% TMCS) and ** μL n-hexane were added into the mixture, which was vortexed vigorously for 2 min and then derivatized at 70 ℃ for 60 min. The samples were placed at ambient temperat ure for 30 min before GC-MS analysis.

注：本英文说明仅供参考使用，请勿直接摘抄到文章里面。如需要请根据自己的标准进行编辑后，再放在文章里面。

1.4 血清

Chemicals

All chemicals and solvents were analytical or HPLC grade. Acetonitrile, methanol were purchased from Thermo Fisher Scientific(Thermo Fisher Scientific, Waltham, MA, USA). Pyridine, n-hexane, methoxylamine hydrochloride（97%）, BSTFA with 1% TMCS were purchased from CNW Technologies GmbH (Düsseldorf, Germany). L-2-chlorophenylalanine was from Shanghai Heng chuang Bio-technology Co., Ltd. (Shanghai, China).

Sample Preparation

Samples stored at -80 °C were thawed at room temperature. ** μL of sample was added to a 1.5 mL Eppendorf tube with ** μL of L-2-chlorophenylalanine (0.3 mg/mL) dissolved in methanol as internal standard, and the tube was vortexed for 10 s. Subsequently, ** μL of ice-cold mixture of methanol and acetonitrile (2/1, vol/vol) was added, and the mixtures were vortexed for 1 min, and the whole samples were extracted by ultrasonic for 10 min in ice-water bath， stored at -20 °C for 30 min. The extract was centrifuged at at 4°C (13,000 rpm) for 10 min. ** mL of supernatant in a glass vial was dried in a freeze concentration centrifugal dryer . QC sample was prepared by mixing aliquot of the all samples to be a pooled sample. An aliquot of the ** μL supernatant was transferred to a glass sampling vial for vacuum-dry at room temperature. And ** μL of 15 mg/mL methoxylamine hydrochloride in pyridine was subsequently added. The resultant mixture was vortexed vigorously for 2 min and incubated at 37 ℃ for 90 mi n. ** μL of BSTFA (with 1% TMCS) and ** μL n-hexane were added into the mixture, which was vortexed vigorously for 2 min and then derivatized at 70 ℃ for 60 min. The samples were placed at ambient temperat ure for 30 min before GC-MS analysis.

1.5 尿液

Chemicals

All chemicals and solvents were analytical or HPLC grade. Methanol, acetonitrile, were purchased from Thermo Fisher Scientific(Thermo Fisher Scientific, Waltham, MA, USA). Pyridine, n-hexane, methoxylamine hydrochloride（97%）, BSTFA with 1% TMCS were purchased from CNW Technologies GmbH (Düsseldorf, Germany). Urease (type C-3) was from Sigma-Aldrich. L-2-chlorophenylalanine was from Shanghai Heng chuang Bio-technology Co., Ltd. (Shanghai, China).

Sample Preparation

** μL aliquot of urine sample was centrifuged at 4°C (13,000 rpm) for 10 min. ** μL aliquot of the supernatant was transferred to a 1.5 mL Eppendorf tube, adding ** μL urease (type C-3, 30 U/10 μL) and incubating for 15 min at 37 °C. The supernatant was spiked with ** μL of L-2-chlorophenylalanine (0.3 mg/mL) dissolved in methanol as internal standard. The mixed solution was extracted with ** μL methanol and acetonitrile (2/1, vol/vol) and vortexed for 30 s, then placed at -20°C for 10 min. The extract was centrifuged at at 4°C (13,000 rpm) for 10 min. ** mL of supernatant in a glass vial was dried in a freeze concentration centrifugal dryer . QC sample was prepared by mixing aliquot of the all samples to be a pooled sample. An aliquot of the ** μL supernatant was transferred to a glass sampling vial for vacuum-dry at room temperature. And ** μL of 15 mg/mL methoxylamine hydrochloride in pyridine was subsequently added. The resultant mixture was vortexed vigorously for 2 min and incubated at 37 °C for 90 mi n. ** μL of BSTFA (with 1% TMCS) and ** μL n-hexane were added into the mixture, which was vortexed vigorously for 2 min and then derivatized at 70 °C for 60 min. The samples were placed at ambient temperat ure for 30 min before GC-MS analysis.

注：本英文说明仅供参考使用，请勿直接摘抄到文章里面。如需要请根据自己的标准进行编辑后，再放在文章里面。

1.6 动物组织

Chemicals

All chemicals and solvents were analytical or HPLC grade. Water, methanol were purchased from Thermo Fisher Scientific(Thermo Fisher Scientific, Waltham, MA, USA). Pyridine, n-hexane, methoxylamine hydrochloride（97%）, BSTFA with 1% TMCS were purchased from CNW Technologies GmbH (Düsseldorf, Germany). Chloroform was from Titan Chemical Reagent Co., Ltd. (Shanghai, China). L-2-chlorophenylalanine was from Shanghai Heng chuang Bio-technology Co., Ltd. (Shanghai, China).

Sample Preparation

** mg accurately weighed sample was transferred to a 1.5 mL Eppendorf tube. Two small steel balls were added to the tube. ** μL of L-2-chlorophenylalanine (0.3 mg/mL) dissolved in methanol as internal standard and ** μL extraction solvent with methanol /water (4/1, vol/vol) were added to each sample. Samples were stored at -20 ℃ for 2 min and then grinded at 60 HZ for 2 min. ** μL of chloroform was added to the samples, and the mixtures were vortexed, than the whole samples were extracted by ultrasonic for 30 min in ice-water bath, then placed at -20°C for 20 min. Samples were centrifuged at 4°C (13,000 rpm) for 10 min . ** μL of supernatant in a glass vial was dried in a freeze concentration centrifugal dryer. QC sample was prepared by mixing aliquot of the all samples to be a pooled sample. An aliquot of the ** μL supernatant was transferred to a glass sampling vial for vacuum-dry at room temperature. And ** μL of 15 mg/mL methoxylamine hydrochloride in pyridine was subsequently added. The resultant mixture was vortexed vigorously for 2 min and incubated at 37 ℃ for 90 mi n. ** μL of BSTFA (with 1% TMCS) and ** μL n-hexane were added into the mixture, which was vortexed vigorously for 2 min and then derivatized at 70 ℃ for 60 min. The samples were placed at ambient temperat ure for 30 min before GC-MS analysis.

注：本英文说明仅供参考使用，请勿直接摘抄到文章里面。如需要请根据自己的标准进行编辑后，再放在文章里面。

2. 样本上机处理

2.1 植物（GC-MS）--7890B-5977A

The derivatived samples were analyzed on an Agilent 7890B gas chromatography system coupled to an Agilent 5977A MSD system (Agilent Technologies Inc., CA, USA). A DB-5MS fused-silica capillary column (30 m × 0.25 mm × 0.25 μm, Agilent J & W Scientific, Folsom, CA, USA) was utilized to separate the derivatives. Helium (> 99.999%) was used as the carrier gas at a constant flow rate of 1 mL / min through the column. The injector temperature was maintained at 260 ℃. Injection volume was ** μL by splitess mode.

The initial oven temperature was 60 °C held at 60 ℃ for 0.5 min, ramped to 125 °C at a rate of 8 °C/min, to 210 °C at a rate of 4 °C/min, to 270 ℃ at a rate of 5 °C/min, to 305 ℃ at a rate of 10 °C/min, and finally held at 305 ℃ for 3 min. The temperature of MS quadrupole, and ion source (electron impact) was set to 150, and 230 ℃, respectively. The collision energy was 70 eV. Mass data was acquired in a full -scan mode (m/z 50-500), and the solvent delay time was set to ** min.

The QCs were injected at regular intervals (every ** samples) throughout the analytical run to provide a set of data from which repeatability can be assessed.

2. 2 动物（GC-MS）--7890B-5977A

The derivatived samples were analyzed on an Agilent 7890B gas chromatography system coupled to an Agilent 5977A MSD system (Agilent Technologies Inc., CA, USA). A DB-5MS fused-silica capillary column (30 m × 0.25 mm × 0.25 μm, Agilent J & W Scientific, Folsom, CA, USA) was utilized to separate the derivatives. Helium (> 99.999%) was used as the carrier gas at a constant flow rate of 1 mL / min through the column. The injector temperature was maintained at 260 ℃. Injection volume was ** μL by splitless mode.

The initial oven temperature was 60 ℃ held at 60 ℃ for 0.5 min, ramped to 125 ℃ at a rate of 8 ℃/min, to 210 ℃ at a rate of 5 ℃/min, to 270 ℃ at a rate of 10 ℃/min, to 305 ℃ at a rate of 20 ℃/min, and finally held at 305 ℃ for 5 min. The temperature of MS quadrupole and ion source (electron impact) was set to 150 and 230 ℃, respectively. The collision energy was 70 eV. Mass spectrometric data was acquired in a full-scan mode (m/z 50-500), and the solvent delay time was set to ** min.

The QCs were injected at regular intervals (every ** samples) throughout the analytical run to provide a set of data from which repeatability could be assessed.

2.3 植物（GC-MS）--7890B-5977B

The derivatived samples were analyzed on an Agilent 7890B gas chromatography system coupled to an Agilent 5977B MSD system (Agilent Technologies Inc., CA, USA). A HP-5MS fused-silica capillary column (30 m × 0.25 mm × 0.25 μm, Agilent J & W Scientific, Folsom, CA, USA) was utilized to separate the derivatives. Helium (> 99.999%) was used as the carrier gas at a constant flow rate of 1 mL / min through the column. The injector temperature was maintained at 260 ℃. Injection volume was ** μL by splitess mode.

The initial oven temperature was 60 °C held at 60 ℃ for 0.5 min, ramped to 125 °C at a rate of 8 °C/min, to 210 °C at a rate of 4 °C/min, to 270 ℃ at a rate of 5 °C/min, to 305 ℃ at a rate of 10 °C/min, and finally held at 305 ℃ for 3 min. The temperature of MS quadrupole, and ion source (electron impact) was set to 150, and 230 ℃, respectively. The collision energy was 70 eV. Mass data was acquired in a full -scan mode (m/z 50-500), and the solvent delay time was set to ** min.

The QCs were injected at regular intervals (every ** samples) throughout the analytical run to provide a set of data from which repeatability can be assessed.

2.4 动物（GC-MS）--7890B-5977B

The derivatived samples were analyzed on an Agilent 7890B gas chromatography system coupled to an Agilent 5977B MSD system (Agilent Technologies Inc., CA, USA). A HP-5MS fused-silica capillary column (30 m × 0.25 mm × 0.25 μm, Agilent J & W Scientific, Folsom, CA, USA) was utilized to separate the derivatives. Helium (> 99.999%) was used as the carrier gas at a constant flow rate of 1 mL / min through the column. The injector temperature was maintained at 260 ℃. Injection volume was ** μL by splitless mode.

The initial oven temperature was 60 ℃ held at 60 ℃ for 0.5 min, ramped to 125 ℃ at a rate of 8 ℃/min, to 210 ℃ at a rate of 5 ℃/min, to 270 ℃ at a rate of 10 ℃/min, to 305 ℃ at a rate of 20 ℃/min, and finally held at 305 ℃ for 5 min. The temperature of MS quadrupole and ion source (electron impact) was set to 150 and 230 ℃, respectively. The collision energy was 70 eV. Mass spectrometric data was acquired in a full-scan mode (m/z 50-500), and the solvent delay time was set to ** min.

The QCs were injected at regular intervals (every ** samples) throughout the analytical run to provide a set of data from which repeatability could be assessed.

注：本英文说明仅供参考使用，请勿直接摘抄到文章里面。如需要请根据自己的标准进行编辑后，再放在文章里面。

3. 数据分析

Data Preprocessing and Statistical Analysis

The obtained GC/MS raw data in .D format were transferred to .abf format via software Analysis Base File Converter for quick retrieval of data. Then, data were imported into software MS-DIAL, which performs peak detection, peak identification, MS2Dec deconvolution, characterization, peak alignment, wave filtering, and missing value interpolation. Metabolite characterization is based on LUG database. A data matrix was derived. The three-dimensional matrix includes: sample information, the name of the peak of each substance, retention time, retention index, mass-to-charge ratio, and signal intensity. In each sample, all peak signal intensities were segmented and normalized according to the internal standards with RSD greater than 0.3 after screening. After the data was normalized, redundancy removal and peak merging were conducted to obtain the data matrix.

The matrix was imported in R to carry out Principle Component Analysis (PCA) to observe the overall distribution among the samples and the stability of the whole analysis process. Orthogonal Partial Least-Squares-Discriminant Analysis (OPLS-DA) and Partial Least-Squares-Discriminant Analysis (PLS-DA) were utilized to distinguish the metabolites that differ between groups. To prevent overfitting, 7-fold cross-validation and 200 Response Permutation Testing (RPT) were used to evaluate the quality of the model.

Variable Importance of Projection (VIP) values obtained from the OPLS-DA model were used to rank the overall contribution of each variable to group discrimination. A two-tailed Student’s T-test was further used to verify whether the metabolites of difference between groups were significant. Differential metabolites were selected with VIP values greater than 1.0 and p-values less than 0.05.
